# Studies Pertaining to the Emerging Cannabinoid Hexahydrocannabinol (HHC)

**DOI:** 10.1021/acschembio.3c00254

**Published:** 2023-08-14

**Authors:** Daniel J. Nasrallah, Neil K. Garg

**Affiliations:** Department of Chemistry Biochemistry, University of California, Los Angeles, California 90095, United States

## Abstract

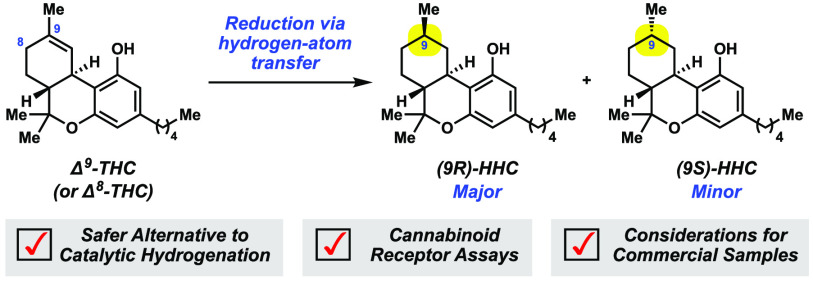

We report studies pertaining to two isomeric hexahydrocannabinols (HHCs), (*9R*)-HHC and (*9S*)-HHC, which are derivatives of the psychoactive cannabinoids Δ^9^- and Δ^8^-THC. HHCs have been known since the 1940s, but have become increasingly available to the public in the United States and are typically sold as a mixture of isomers. We show that (*9R*)-HHC and (*9S*)-HHC can be prepared using hydrogen-atom transfer reduction, with (*9R*)-HHC being accessed as the major diastereomer. In addition, we report the results of cannabinoid receptor studies for (*9R*)-HHC and (*9S*)-HHC. The binding affinity and activity of isomer (*9R*)-HHC are similar to that of Δ^9^-THC, whereas (*9S*)-HHC binds strongly in cannabinoid receptor studies but displays diminished activity in functional assays. This is notable, as our examination of the certificates of analysis for >60 commercially available HHC products show wide variability in HHC isomer ratios (from 0.2:1 to 2.4:1 of (*9R*)-HHC to (*9S*)-HHC). These studies suggest the need for greater research and systematic testing of new cannabinoids. Such efforts would help inform cannabis-based policies, ensure the safety of cannabinoids, and potentially lead to the discovery of new medicines.

## Introduction

**Figure 1:**
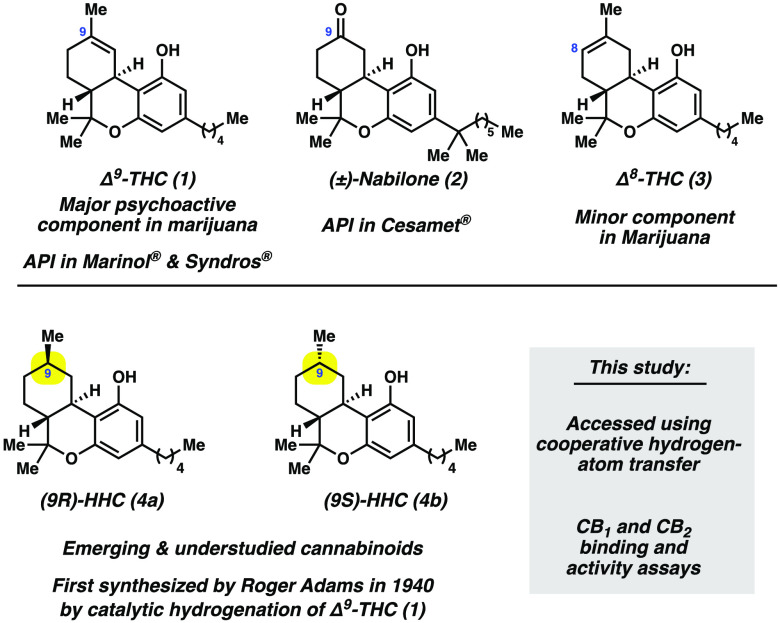
Cannabinoids **1**–**3** and emerging cannabinoids HHCs **4a** and **4b**.

The cannabis industry has undergone remarkable evolution in recent years. Despite marijuana, cannabis having ≥0.3% (weight/weight) of Δ^9^-*trans*-tetrahydrocannabinol (Δ^9^-THC, see compound **1**, Figure 1),^1^ being illegal in many parts of the world and stigmatized for decades, many states in the United States (U.S.) have now legalized or decriminalized the use of marijuana-based products.^2^ Similarly, there has been an increase in U.S. Federal legislation,^2,3^ with a particular focus on accelerating the pace of research needed to address numerous challenges in the field.^4−6^

One major contemporary challenge involves the availability of derivatives of Δ^9^-THC (**1**, Figure 1),^7^ which is the primary active component of marijuana that is associated with intoxication. However, from a therapeutic standpoint, **1** is also the active pharmaceutical ingredient (API) in the FDA-approved drugs Marinol and Syndros. These drugs are used to treat nausea and vomiting caused by cancer chemotherapy, in addition to loss of appetite and weight loss in patients with HIV/AIDS. Whereas **1** on its own is a Schedule 1 substance, Marinol is a Schedule 3 substance, and Syndros is a Schedule 2 substance.

Not surprisingly, analogs of **1** have similarly become highly sought after for both medicinal and nonmedicinal purposes.^8^ THC derivatives **2** and **3** provide illustrative examples. Nabilone (**2**) is sold as a racemate under the trade name Cesamet and is used for the treatment of chronic pain (in Canada). It is also FDA-approved for chemotherapy-induced vomiting or nausea (in the U.S.). A contrasting example is Δ^8^-*trans*-tetrahydrocannabinol (Δ^8^-THC, **3**), a minor constituent of cannabis with a structure similar to **1**. Δ^8^-THC (**3**) has become commonly available to the public in many states, both with and without marijuana legalization, yet remains non-FDA-approved, unregulated, and generally understudied.^9^

The present study focuses on related cannabinoids called hexahydrocannabinols (HHCs).^10,11^ Despite being relatively understudied since the first synthesis by Adams in 1940,^12^ these compounds are now becoming increasingly widespread and can be purchased in some states in the U.S. Conflicting views regarding the federal legality of HHCs exist. One perspective is that HHCs are legal as a result of the U.S. 2018 Farm Bill. On the other hand, the U.S. Drug Enforcement Administration (DEA) has informed us that they consider HHCs of the type described herein Schedule 1 substances.

When HHCs are accessed synthetically from Δ^9^-THC (**1**) or Δ^8^-THC (**3**), two diastereomers can form: (*9R*)-HHC **4a** and (*9S*)-HHC **4b** (Figure 1). The *9R* isomer, **4a**, is sometimes referred to as the “methyl equatorial” isomer of HHC in the literature, whereas the *9S* isomer, **4b**, is sometimes referred to as the “methyl axial” isomer. These HHC diastereomers vary based on the stereochemistry at C9. It is expected that different properties and biological effects exist for the two diastereomers. The ratio of isomers **4a** and **4b** within commercially available HHC varies significantly, as will be discussed further in this Letter, presumably based on the method of production and purification. Most commonly, **4a** and **4b** are prepared using catalytic hydrogenation,^12−15^ which yields a mixture of isomers with low selectivity. In addition, fires, runaway reactions, and explosions are well-known dangers associated with catalytic hydrogenation and such dangers can vary based on the conditions employed.^16−19^ Lastly, trace heavy metals (e.g., Pt or Pd) may remain after catalytic hydrogenation due to leaching or dissolution. The extent to which trace metals remain can vary based on the catalyst employed, the reaction conditions used, as well as the exact purification methods.^20,21^ The presence of residual heavy metals bears significant toxicity concerns.

Limited biological studies of HHCs **4a** and **4b** are available in the literature.^22−31^ The psychoactive effects of **4a** and **4b** have been demonstrated in rabbits^24^ and nonhuman primates,^22,23,25^ using either individual isomers or mixtures. With regard to therapeutic potential, studies have shown that HHCs **4a** and **4b** may be valuable leads for the treatment of colon cancer^26^ and ocular hypotony.^27^ One recent study shows promising cardiac safety and cytotoxicity profiles for the mixture of HHC isomers using in vitro assays.^28^ There is also one report regarding the in vitro binding affinity of (±)-**4a** in human cannabinoid receptors.^30^ Systematic in vitro assay data showing potency or binding affinity of enantioenriched **4a** or **4b** to the cannabinoid receptors type 1 or 2 (CB_1_ or CB_2_), have yet to be published. Of note, cannabinoids that bind to either the CB_1_ or CB_2_ receptor may be associated with both adverse effects and therapeutic potential, depending on a variety of factors.^32,33^ Cannabinoids that bind to the CB_1_ receptor are also commonly associated with intoxicating effects. Individually investigating the biological characteristics of HHC isomers **4a** and **4b** would provide insight into their therapeutic potential.

With the aforementioned considerations, we sought to (a) develop a means to synthesize both isomers of HHC that avoids the use of potentially dangerous catalytic hydrogenation conditions and toxic heavy metals and (b) establish their affinity and activity at human CB_1_ and CB_2_ receptors relative to Δ^9^-THC (**1**). Here, we report the use of cooperative hydrogen-atom transfer (HAT) to convert Δ^9^-THC (**1**) and Δ^8^-THC (**3**) to HHCs **4a** and **4b**.^34^ In contrast to results obtained by using catalytic hydrogenation, **4a** is formed as the major product using the HAT protocol. Additionally, we demonstrate via biological investigation that **4a** and **4b** possess significantly different binding affinities and potency, with **4a** exhibiting similar activity compared with Δ^9^-THC (**1**). Finally, we examine the certificates of analyses for >60 commercially available HHC products, which show wide variability in HHC isomer ratios. These studies are expected to prompt further scientific investigation of HHCs, and also underscore the importance of performing such synthetic and biochemical studies as new cannabinoids inevitably emerge.

### Reduction of Δ^9^-THC (**1**) and Δ^8^-THC (**3**)

**Scheme 1:**
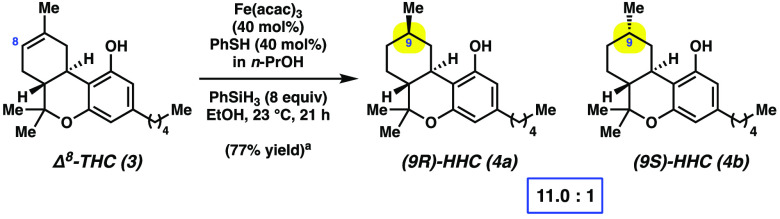
HAT Reduction of Δ^8^-THC (**3**) To Give HHCs **4a** and **4b** Yield reflects the average of two isolation experiments.

To initiate our studies, we investigated the reduction of Δ^9^-THC (**1**) to afford HHCs **4a** and **4b** (see Table 1). We first attempted catalytic hydrogenation conditions, since literature data did not consistently report diastereoselectivities or yields. We began with the use of PtO_2_, which Adams had demonstrated in the 1940s for the reduction of **1**.^12^ This gave roughly equimolar amounts of **4a** and **4b** (entry 1 in Table 1). Similar results were seen when Pt/C was used (entry 2 in Table 1). Although the use of Rh/C proved to be ineffective (entry 3 in Table 1), hydrogenation using Pd/C delivered **4a** and **4b** in a ratio of 1 to 3.9, respectively (entry 4 in Table 1). We then examined homogeneous hydrogenation conditions that were not known for the reduction of **1** in the literature. The use of Ir catalyst (Crabtree’s catalyst) proved less effective (entry 5 in Table 1), while Rh catalyst (Wilkinson’s catalyst) was unsuccessful (entry 6 in Table 1). The reduction with diimide **5** also proved unproductive (entry 7 in Table 1). Stoichiometric metal hydride conditions gave low yields (entry 8 in Table 1).^35^ Lastly, we evaluated HAT conditions, which are attractive due to the avoidance of pyrophoric reagents and high levels of thermodynamically controlled diastereoselectivity.^36^ Such conditions had not been reported for the reduction of **1**. The Fe-based protocol reported by West was especially attractive,^37^ as the necessary reagents are commercially available and iron is considered a metal of minimal concern.^20^ Employment of the West conditions gave good diastereoselectivity (entry 9 in Table 1) but a low yield. By increasing the loading of reagents and adding them portionwise over two additions, the combined yield of **4a** and **4b** increased to 74%, with a **4a**:**4b** ratio of 9.5:1 (entry 10 in Table 1). It is notable that the HAT conditions provide the highest selectivity and favor the formation of isomer **4a**. In consideration of the results described in the subsequent section pertaining to biological assays, the observed preference for **4a** proved to be a fortuitous result. We also examined the HAT reduction of the Δ^9^-THC (**1**) isomer Δ^8^-THC (**3**). As shown in Scheme 1, treatment of **3** under our previously optimized conditions afforded HHCs **4a** and **4b** in 77% yield. Of note, the **4a**:**4b** ratio was 11.0 to 1, respectively, which reflects comparable selectivity to the diastereoselectivity observed when Δ^9^-THC (**1**) is employed. These results show that either THC isomer (i.e., **1** or **3**) can be used in the reduction.

**Table 1:**
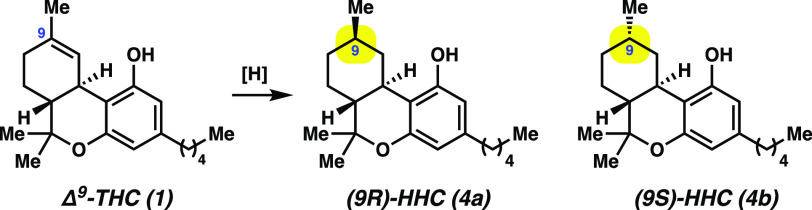

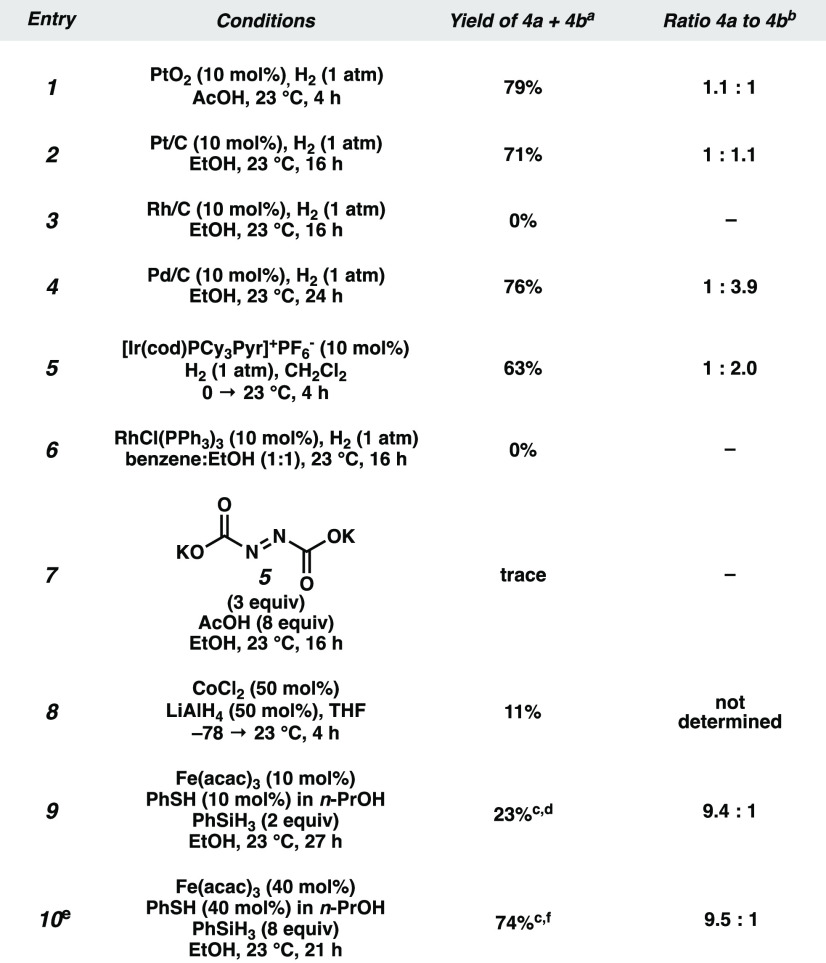
Reduction Studies of Δ^9^-THC (**1**) To Furnish HHCs **4a** and **4b**

a Isolated yields.

b Ratios determined from isolated material using ^1^H NMR.

c Yields reflect the average of two isolation experiments.

d 38% yield of recovered **1**, average from two isolation experiments.

e 20 mol % of Fe(acac)_2_, 20 mol % of PhSH, and 4 equiv PhSiH_3_ were initially added portionwise, followed by the second portion after 17 h.

f 10% yield of recovered **1**, average from two isolation experiments.

**Figure 2:**
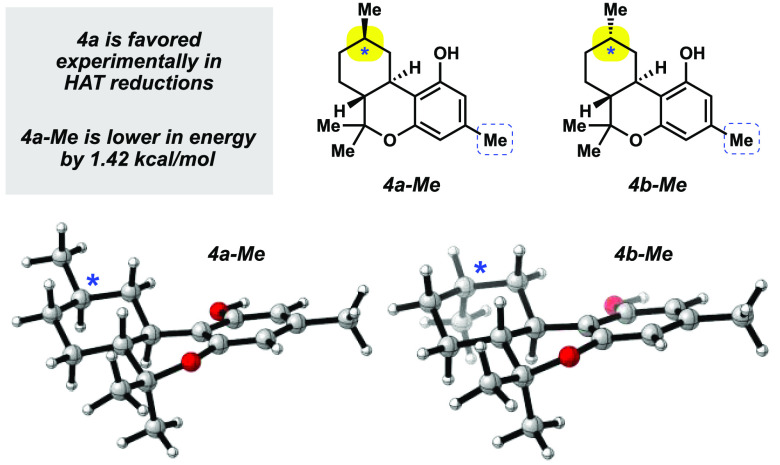
Lowest energy conformers for **4a-Me** and **4b-Me**. Structures are displayed by using CYLview.

In order to rationalize the diastereoselectivity in the HAT reduction of **1** and **3**, we considered the relative energies of **4a** and **4b**. A 1989 study by Reggio and co-workers suggested that **4a** is energetically favorable,^38^ but computations using higher levels of theory now accessible have not been performed. As such, we conducted density functional theory (DFT) calculations using ωB97X-D (6-31G*) for **4a-Me** and **4b-Me**, which are the simplified structures (i.e., methyl in place of pentyl) shown in Figure 2. Following conformational searching, the lowest ground-state energies were compared. Of note, **4a-Me** was found to be thermodynamically favored by 1.42 kcal/mol. This difference in energy corresponds to a diastereomeric ratio (dr) of ∼10:1, which is consistent with our experimental observations. In accordance with general mechanistic considerations for HAT reductions,^36^ thermodynamic control is presumably operative in the reductions of **1** or **3** to favor formation of the product bearing an equatorial methyl group (i.e., **4a**, analogous to computed structure **4a-Me**).

### CB_1_ and CB_2_ Receptor Studies

As discussed earlier, two receptors, CB_1_ and CB_2_, are important for assessing the biological activities of cannabinoids. Interactions with CB_1_ or CB_2_ can be useful for therapeutic purposes.^32,33^ Given that the individual activities of **4a** and **4b** in human CB_1_ and CB_2_ receptor assays are not described in the literature, we sought to study this further. Pure synthetic samples of **4a** and **4b** were prepared (>19:1 dr) for use in cannabinoid receptor studies. Given the relative product distribution of the different reduction protocols, **4a** was accessed using HAT reduction of **1**, whereas **4b** was prepared using catalytic hydrogenation of **1** (see the SI for details). Biological assays were performed by Eurofins Discovery. A radioligand binding assay was used to determine *K*_*i*_ and IC_50_ values.^39,40^ A G-protein coupled receptor (GPCR) functional assay was used to study potency and determine EC_50_ values.^41^ Both assays use human cannabinoid receptors with the appropriate reference standards (see the SI for details). Δ^9^-THC (**1**), a known partial agonist for both CB_1_ and CB_2_, was simultaneously evaluated to provide a point of comparison under the same assay conditions.^42^

**Figure 3:**
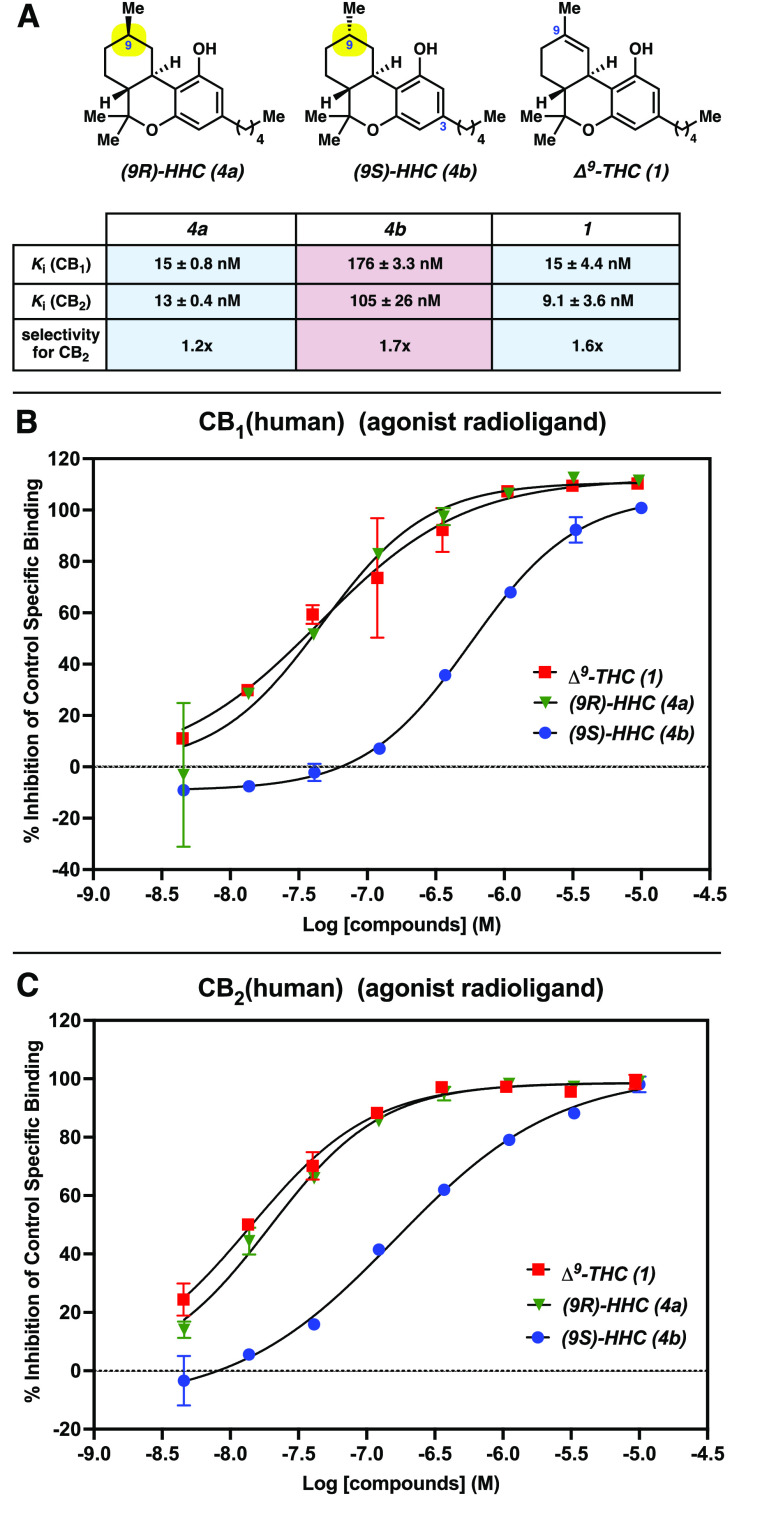
(A) Summary of radioligand binding affinity studies; error values represent the standard deviation. (B) Plotted inhibition of binding for human CB_1_ cannabinoid receptor in transfected Chem-1 cell lysate after treatment with **1**, **4a**, and **4b**. (C) Plotted inhibition of binding for human CB_2_ cannabinoid receptor in transfected CHO cell lysate after treatment with **1**, **4a**, and **4b**. For all experiments, data represent two replicate experiments, with error bars showing standard deviation (error bars omitted for clarity if the range is smaller than the data symbol), and the *Y*-intercept was constrained to zero, unless otherwise noted (see the SI for details).

**Figure 4:**
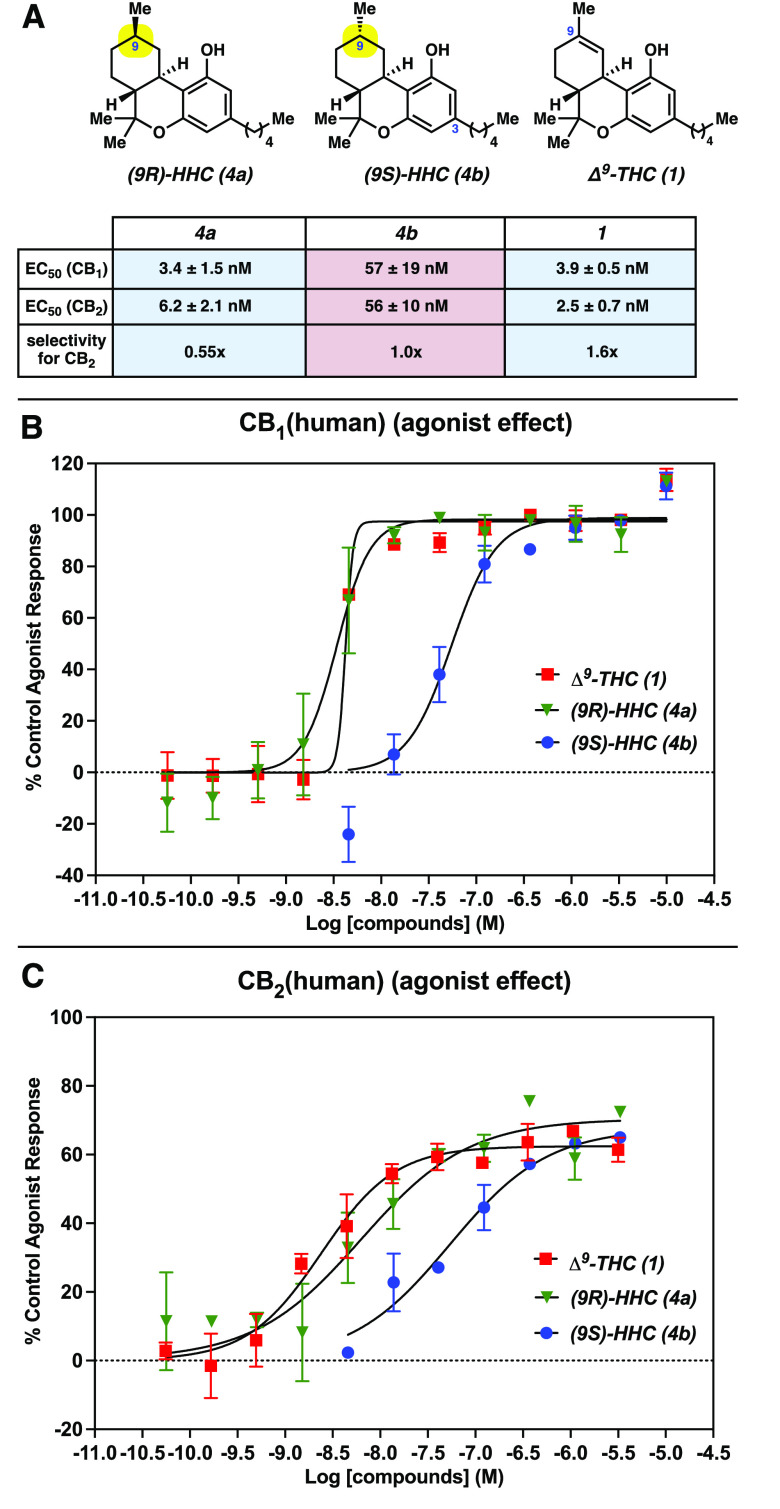
(A) Summary of functional activity studies; error values represent the standard deviation. (B) Plotted response of human CB_1_ receptor expressed in transfected CHO cells after treatment with **1**, **4a**, and **4b**, determined by measuring their effects on cAMP concentration. (C) Plotted response of human CB_2_ receptor expressed in transfected CHO cells after treatment with **1**, **4a**, and **4b**, determined by measuring their effects on cAMP concentration. For all experiments, data represent two replicate experiments, with error bars showing the standard deviation (error bars omitted for clarity if the range is smaller than the data symbol) and the *Y*-intercept was constrained to zero unless otherwise noted (see the SI for details).

The CB_1_ binding assay was conducted with cellular lysates of Chem-1 cells transfected with human CB_1_ cannabinoid receptor. Displacement of radio-labeled [^3^H]CP 55940^43^ by the tested compounds measured specific binding and WIN 55212-2^44^ was used to determine nonspecific binding. The CB_2_ binding assay was conducted with cellular lysates of CHO cells transfected with the human CB_2_ cannabinoid receptor. Displacement of radio-labeled [^3^H]WIN 55212-2 by the tested compounds measured specific binding and WIN 55212-2 was used to determine nonspecific binding. Both **4a** (*K*_*i*_ = 15 nM at CB_1_ and 13 nM at CB_2_) and **4b** (*K*_*i*_ = 176 nM at CB_1_ and 105 nM at CB_2_) bind to the CB_1_ and CB_2_ receptors with nanomolar affinity in the radioligand assay (Figure 3A). A comparison of the binding of each individual diastereomer to the CB_1_ and CB_2_ receptors shows minimal selectivity (1.2–1.7x for CB_2_) for binding to one receptor over the other. However, as shown in Figures 3B and 3C, **4a** (green triangle) binds both receptors with an affinity an order of magnitude higher than that of **4b** (blue circle), demonstrating stronger binding of the (*9R*)-HHC **4a** diastereomer. Of note, the binding affinity of **4a** (green triangle) is similar to that of Δ^9^-THC (**1**, red square) for both cannabinoid receptors.

The CB_1_ and CB_2_ functional assays were conducted using CHO cells transfected with human CB_1_ and CB_2_ cannabinoid receptors, respectively. Efficacy of the tested compounds was determined by measuring changes in cAMP concentrations relative to controls using homogeneous time-resolved fluorescence^45^ (HTRF). In the functional assay, **4a** (EC_50_ = 3.4 nM at CB_1_ and 6.2 nM at CB_2_) and **4b** (EC_50_ = 57 nM at CB_1_ and 56 nM at CB_2_) demonstrated excitatory activity at the CB_1_ and CB_2_ receptors (Figure 4A). Of note, **4a** shows modest selectivity (1.8x) for CB_1_ whereas there is no significant selectivity of **4b** between the CB_1_ or CB_2_ receptors. Figures 4B and 4C show that **4a** (green triangle) has 17- and 9-fold increases in potency, compared to **4b** (blue circle) for CB_1_ and CB_2_, respectively. This activity of **4a** (green triangle) is similar to that of Δ^9^-THC (**1**, red square). However, both HHCs **4a** and **4b**, such as Δ^9^-THC (**1**), are partial agonists of both receptors. Collectively, the results of the binding and functional assays demonstrate significant biological differences between HHC diastereomers **4a** and **4b**. These two diastereomers differ only in the directionality of the C9 methyl group, highlighting the effect of subtle structural modifications to the cannabinoid scaffold. These data also illustrate the need to understand the activity of individual compounds instead of mixtures, especially as new cannabinoids become available.

### Chemical Composition of HHC Products

Given the different biological profiles of HHCs **4a** and **4b** described above, it is important to understand the chemical composition of recreational HHC products that are becoming increasingly widespread. We examined the online certificates of analysis for >60 HHC-containing products (see the SI for details). For 15 samples, the **4a**:**4b** ratios were not provided. For the others, the **4a**:**4b** ratios ranged from 0.2:1 to 2.4:1 (average of 1.4:1). As such, roughly 15%–70% of a given sample’s HHC content is composed of the more potent isomer **4a**. This wide variability in chemical composition and the potential presence of heavy metals may have public health consequences.

Because catalytic hydrogenation of Δ^9^-THC (**1**) or Δ^8^-THC (**3**) is typically the final step in the synthesis of HHCs **4a** and **4b**, we questioned if consumer products had been analyzed for the heavy metals typically used for catalysis (i.e., Pd or Pt).^20,21^ Numerous cannabis analytical laboratories were contacted in different states. Although testing for arsenic, mercury, lead, and cadmium is typically available as required by cannabis laws in most or all states, we learned that testing for Pd or Pt is rarely requested or offered at most cannabis analytical testing facilities. Several laboratories reiterated that such testing is not required. It was not possible for us to acquire and test commercial samples of HHC for heavy metals due to DEA regulations. We encourage that such testing be performed by HHC producers to ensure consumer safety, perhaps drawing from well-established practices for pharmaceutical production.

## Conclusions

We have performed studies pertaining to HHCs **4a** and **4b**, which are emerging cannabinoids first synthesized in the 1940s. Our current study shows that HHCs **4a** and **4b** can be prepared using HAT reduction of Δ^9^-THC (**1**) or Δ^8^-THC (**3**), thus providing an alternative to classic hydrogenation conditions. This is notable, because the HAT reduction protocols provide **4a** as the major diastereomer, whereas classical hydrogenation conditions are less selective, leading to mixtures of isomers being available to consumers. Moreover, the traditional catalytic hydrogenation protocols, which bear significant safety risks, can be avoided. In addition, we have performed cannabinoid receptor studies of each diastereomer with Δ^9^-THC (**1**) as a control in the same assays. Both isomers **4a** and **4b** were shown to have partial CB_1_ and CB_2_ receptor agonist activity, such as Δ^9^-THC (**1**). However, isomer **4a**, accessed as the major diastereomer in the HAT reduction protocol, binds with higher affinity (*K*_*i*_ = 15 and 13 nM at CB_1_ and CB_2_, respectively) and displays good activity in the functional assay (EC_50_ = 3.4 and 6.2 nM at CB_1_ and CB_2_, respectively); the activity of isomer **4a** nearly matches that of Δ^9^-THC (**1**). This study demonstrates how minor modifications to the Δ^9^-THC (**1**) scaffold can lead to significant and varying differences in biological activity. Furthermore, the results of this study illustrate the importance of conducting assays on singular compounds in order to draw conclusions about how changes to the three-dimensional space in derivatives affect both their binding and potency. Lastly, this study shows that the method of Δ^9^-THC (**1**) reduction greatly affects the diastereomeric ratio of the HHC products formed. HAT provides an alternative method to access the more active (*9R*)-HHC isomer (**4a**) and could enable further biological evaluation. As highlighted in recent articles,^46−48^ further studies of HHCs are desirable.

Although our scientific findings should not be used on their own to create federal or state policies, this study prompts many future directions that should be considered. For example, it would benefit society to increase the pace of chemical and biochemical-based research in the cannabinoid field while maintaining scientific rigor. New cannabinoids are emerging, and increased fundamental research is needed to characterize their in vitro and in vivo pharmacology and assess psychoactivity, therapeutic potential, and safety. In the U.S., this need has already been recognized by the federal government with recent efforts aimed at helping researchers secure faster approval to perform their research. However, more attention could be paid to the rising availability of new cannabis-derived compounds and analogs.^2,3^

With regard to specific research areas that require attention, we offer our view that organic and medicinal chemistry can play an important role in the cannabinoid research space. Historically, efforts by organic chemists to impact cannabinoid research have been instrumental. In this rapidly evolving climate, further engagement should be encouraged. Chemists can contribute by preparing new compounds using synthesis, semisynthesis, or biocatalysis, performing careful analyses of chemical composition and contaminants, conducting assays, investigating the mechanisms of action, performing structure-activity relationship studies, exploring receptor binding using computational studies, and ultimately engaging in advanced studies, such as clinical trials. Such efforts could help inform cannabis-based policies and regulations, ensure the safe and fair use of cannabinoids, and ultimately lead to the discovery of new medicines.

## Supporting Information Available

Detailed experimental procedures, compound characterization data, description of biological assays (PDF)

## References

[ref1] Sacco L. N. Evolution of Marijuana as a Controlled Substance and the Federal-State Policy Gap. Congressional Research Service, April 7, 2022. Available via the Internet at: https://crsreports.congress.gov/product/pdf/R/R44782 (accessed April 13, 2023).

[ref2] Lampe J. R. Recent Developments in Marijuana Law. Congressional Research Service, Dec. 6, 2022. Available via the Internet at: https://crsreports.congress.gov/product/pdf/LSB/LSB10859 (accessed April 13, 2023).

[ref3] Erickson B. E. Cannabis research bill clears U.S. Congress. Chem. Eng. News, 2022. Available via the Internet at: https://cen.acs.org/biological-chemistry/natural-products/Cannabis-research-bill-clears-US/100/i43 (accessed April 13, 2023).

[ref4] Wadman M. New U.S. law aims to light up medical research on cannabis. Science 2022, 378, 1035 10.1126/science.adg1903. 36480616

[ref5] Devitt T.; Epstein P.; Phillips N.; Devitt-Lee A. Pandora’s box the dangers of a national, unregulated, hemp-derived intoxicating cannabinoid market. California Cannabis Industry Association, 2022. Available via the Internet at: https://www.projectcbd.org/sites/projectcbd/files/downloads/white-paper_hemp_2022-10-18.pdf (accessed April 13, 2023). [White paper.]

[ref6] Legal weed, broken promises: A Times series on the fallout of legal pot in California. Los Angeles Times. Sept. 8, 2022, updated Dec. 29, 2022. Available via the Internet at: https://www.latimes.com/california/story/2022-09-08/a-series-on-the-fallout-of-legal-weed-in-california (accessed April 13, 2023).

[ref7] Iversen L. The pharmacology of delta-9-Tetrahydrocannabinol (THC). In The Science of Marijuana, 3rd Edition; Oxford University Press, 2018; p 22-C2.F7.

[ref8] Willner N. The Controlled Substances Act leaves pathway for intoxicating hemp-derived cannabinoids. MJBizDaily, Feb. 1, 2022. Available via the Internet at: https://mjbizdaily.com/the-controlled-substances-act-leaves-pathway-for-intoxicating-hemp-derived-cannabinoids/ (accessed April 13, 2023).

[ref9] Erickson B. E. Delta-8-THC craze concerns chemists. In Chem. Eng. News, 2021. Available via the Internet at: https://cen.acs.org/biological-chemistry/natural-products/Delta-8-THC-craze-concerns/99/i31 (accessed Jan. 14, 2023).

[ref10] Qureshi M. N.; Kanwal F.; Siddique M.; Inayat-ur-Rahman A. M. Estimation of biologically active cannabinoids in cannabis indica by gas chromatography-mass spectrometry (GC-MS). World Appl. Sci. J. 2012, 19, 918–923. 10.5829/idosi.wasj.2012.19.07.1922.

[ref11] Collins A. C.; Ramirez G. A.; Tesfatsion T. T.; Ray K. P.; Cruces W. Characterization of hexahydrocannabinol (HHC) diastereomers, and hexahydrocannabidiol (H4CBD) diastereomers using NMR, HPLC, and GC-MS. Res. Square 2022, 1, 10.21203/rs.3.rs-2322468/v1.

[ref12] Adams R.; Pease D. C.; Cain C. K.; Clark J. H. Structure of cannabidiol. VI. Isomerization of cannabidiol to tetrahydrocannabinol, a physiologically active product. Conversion of cannabidiol to cannabinol. J. Am. Chem. Soc. 1940, 62, 2402–2405. 10.1021/ja01866a040.

[ref13] Adams R.; Cain C. K.; McPhee W. D.; Wearn R. B. Structure of cannabidiol. XII. Isomerization to tetrahydrocannabinols. J. Am. Chem. Soc. 1941, 63, 2209–2213. 10.1021/ja01853a052.

[ref14] Adams R. Marihuana active compounds. U.S. Patent No. US2419937A, March 27, 1944.

[ref15] Gaoni Y.; Mechoulam R. Hashish – VII The isomerization of cannabidiol to tetrahydrocannabinols. Tetrahedron 1966, 22, 1481–1488. 10.1016/S0040-4020(01)99446-3.

[ref16] Solis N. 2 dead after explosive fire at suspected hemp lab in Canoga Park. Los Angeles Times, Oct. 19, 2021. Available via the Internet at: https://www.google.com/amp/s/www.latimes.com/california/story/2021-10-19/2-dead-after-fire-at-suspected-pot-warehouse-in-canoga-park%3f_amp=true (accessed Jan. 11, 2023).

[ref17] Ruscitto A. What is HHC? Cannabis Business Times. Feb. 9, 2022. Available via the Internet at: https://www.cannabisbusinesstimes.com/article/what-is-hexahydrocannabinol-hhc-hemp-derived-cannabinoid-thc/ (accessed Jan. 11, 2023).

[ref18] Chandra T.; Zebrowski J. P. Hazards associated with laboratory scale hydrogenations. J. Chem. Health Saf. 2016, 23, 16–25. 10.1016/j.jchas.2015.10.019.

[ref19] Fannes C.; Verbruggen S.; Janssen B.; Egle B. Influence of solvents and additives on the pyrophoricity of palladium on carbon catalyst after hydrogenation. Org. Process Res. Dev. 2021, 25, 2438–2441. 10.1021/acs.oprd.1c00190.

[ref20] Raghuram P.; Soma Raju I. V.; Sriramulu J. Heavy metals testing in active pharmaceutical ingredients: an alternate approach. Pharmazie 2010, 65, 15–18. 20187573

[ref21] Miyamoto H.; Sakumoto C.; Takekoshi E.; Maeda Y.; Hiramoto N.; Itoh T.; Kato Y. Effective method to remove metal elements from pharmaceutical intermediates with polychelated resin scavenger. Org. Process Res. Dev. 2015, 19, 1054–1061. 10.1021/acs.oprd.5b00161.

[ref22] Edery H.; Grunfeld Y.; Ben-Zvi Z.; Mechoulam R. Structural requirements for cannabinoid activity. Ann. N.Y. Acad. Sci. 1971, 191, 40–53. 10.1111/j.1749-6632.1971.tb13985.x.

[ref23] Mechoulam R.; Lander N.; Varkony T. H.; Kimmel I.; Becker O.; Ben-Zvi Z.; Edery H.; Porath G. Stereochemical requirements for cannabinoid activity. J. Med. Chem. 1980, 23, 1068–1072. 10.1021/jm00184a002. 7420350

[ref24] Consroe P.; Martin A. R.; Fish B. S. Use of a potential rabbit model for structure-behavioral activity studies of cannabinoids. J. Med. Chem. 1982, 25, 596–599. 10.1021/jm00347a021. 7086846

[ref25] Edery H.; Porath G.; Mechoulam R.; Lander N.; Srebnik M.; Lewis N. Activity of novel aminocannabinoids in baboons. J. Med. Chem. 1984, 27, 1370–1373. 10.1021/jm00376a029. 6541257

[ref26] Thapa D.; Babu D.; Park M.-A.; Kwak M.-K.; Lee Y.-R.; Kim J. M.; Kwon T. K.; Kim J.-A. Induction of p53-independent apoptosis by a novel synthetic hexahydrocannabinol analog is mediated via Sp1-dependent NSAID-activated gene-1 in colon cancer cells. Biochem. Pharmacol. 2010, 80, 62–71. 10.1016/j.bcp.2010.03.008. 20230799

[ref27] Elsohly M. A.; Harland E. C.; Benigni D. A.; Waller C. W. Cannabinoids in glaucoma II: The effect of different cannabinoids on intraocular pressure of the rabbit. Curr. Eye Res. 1984, 3, 841–850. 10.3109/02713688409000797. 6329602

[ref28] Collins A.; Tesfatsion T.; Ramirez G.; Ray K.; Cruces W. Nonclinical in vitro safety assessment summary of hemp derived (R/S)-hexahydrocannabinol ((R/S)-HHC). Cannabis Sci. Technol. 2022, 5, 23–27.

[ref29] Harvey D. J.; Brown N. K. Comparative in vitro metabolism of the cannabinoids. Pharmacol., Biochem. Behav. 1991, 40, 533–540. 10.1016/0091-3057(91)90359-A. 1806943

[ref30] Sanchez Montero J. M.; Agis-Torres A.; Solano D.; Söllhuber M.; Fernandez M.; Villaro W.; Gómez-Cañas M.; García-Arencibia M.; Fernández-Ruiz J.; Egea J.; Martín M. I.; Girón R. Analogues of cannabinoids as multitarget drugs in the treatment of Alzheimer’s disease. Eur. J. Pharmacol. 2021, 895, 173875 10.1016/j.ejphar.2021.173875. 33460612

[ref31] Nikas S. P.; Alapafuja S. P.; Papanastasiou I.; Paronis C. A.; Shukla V. G.; Papahatjis D. P.; Bowman A. L.; Halikhedkar A.; Han X.; Makriyannis A. Novel 1′,1′-chain substituted hexahydrocannabinols: 9βHydroxy-3-(1-hexyl-cyclobut-1-yl)-hexahydrocannabinol (AM2389) a highly potent cannabinoid receptor 1 (CB_1_) agonist. J. Med. Chem. 2010, 53, 6996–7010. 10.1021/jm100641g. 20925434 PMC3650853

[ref32] An D.; Peigneur S.; Hendrickx L. A.; Tytgat J. Targeting cannabinoid receptors: Current status and prospects of natural products. Int. J. Mol. Sci. 2020, 21, 5064 10.3390/ijms21145064. 32709050 PMC7404216

[ref33] Lutz B. Neurobiology of cannabinoid receptor signaling. Dialogues Clin. Neurosci. 2020, 22, 207–222. 10.31887/DCNS.2020.22.3/blutz. 33162764 PMC7605026

[ref34] Kattamuri P. V.; West J. G. Cooperative hydrogen atom transfer: From theory to applications. Synlett 2021, 32, 1179–1186. 10.1055/a-1463-9527.

[ref35] Ashby E. C.; Lin J. J. Selective reduction of alkenes and alkynes by the reagent lithium aluminum hydride-transition-metal halide. J. Org. Chem. 1978, 43, 2567–2572. 10.1021/jo00407a004.

[ref36] Green S. A.; Crossley S. W. M.; Matos J. L. M.; Vaśquez–Ceśpedes S.; Shevick S. L.; Shenvi R. A. The high chemofidelity of metal-catalyzed hydrogen atom transfer. Acc. Chem. Res. 2018, 51, 2628–2640. 10.1021/acs.accounts.8b00337. 30406655 PMC6248883

[ref37] Kattamuri P. V.; West J. G. Hydrogenation of alkenes via cooperative hydrogen atom transfer. J. Am. Chem. Soc. 2020, 142, 19316–19326. 10.1021/jacs.0c09544. 33119986

[ref38] Reggio P. H.; Greer K. V.; Cox S. M. The importance of the Orientation of the C9 Substituent to cannabinoid activity. J. Med. Chem. 1989, 32, 1630–1635. 10.1021/jm00127a038. 2738895

[ref39] Munro S.; Thomas K. L.; Abu-Shaar M. Molecular characterization of a peripheral receptor for cannabinoids. Nature 1993, 365, 61–65. 10.1038/365061a0. 7689702

[ref40] Rinaldi-Carmona M.; Calandra B.; Shire D.; Bouaboula M.; Oustric D.; Barth F.; Casellas P.; Ferrara P.; Le Fur G. Characterization of two cloned human CB_1_ cannabinoid receptor isoforms. J. Pharmacol. Exp. Ther. 1996, 278, 871–878. 8768742

[ref41] Felder C. C.; Joyce K. E.; Briley E. M.; Mansouri J.; Mackie K.; Blond O.; Lai Y.; Ma A. L.; Mitchell R. L. Comparison of the pharmacology and signal transduction of the human cannabinoid CB_1_ and CB_2_ receptors. Mol. Pharmacol. 1995, 48, 443–450. 7565624

[ref42] Howlett A. C.; Barth F.; Bonner T. I.; Cabral G.; Casellas P.; Devane W. A.; Felder C. C.; Herkenham M.; Mackie K.; Martin B. R.; Mechoulam R.; Pertwee R. G. International Union of Pharmacology. XXVII. Classification of cannabinoid receptors. Pharmacol. Rev. 2002, 54, 161–202. 10.1124/pr.54.2.161. 12037135

[ref43] Devane W. A.; Dysarz F. A.; Johnson M. R.; Melvin L. S.; Howlett A. C. Determination and characterization of a cannabinoid receptor in rat brain. Mol. Pharmacol. 1988, 34, 605–613. 2848184

[ref44] D’Ambra T. E.; Estep K. G.; Bell M. R.; Eissenstat M. A.; Josef K. A.; Ward S. J.; Haycock D. A.; Baizman E. R.; Casiano F. M.; Beglin N.; Chippari S. M.; Grego J. D.; Kullnig R. K.; Daley G. T. Conformationally restrained analogs of pravadoline: nanomolar potent, enantioselective, (aminoalkyl) indole agonists of the cannabinoid receptor. J. Med. Chem. 1992, 35, 124–135. 10.1021/jm00079a016. 1732519

[ref45] Degorce F.; Card A.; Soh S.; Trinquet E.; Knapik G. P.; Xie B. HTRF: A technology tailored for drug discovery – A review of theoretical aspects and recent applications. Curr. Chem. Genomics 2009, 3, 22–32. 10.2174/1875397300903010022. 20161833 PMC2802762

[ref46] Ujváry I. Hexahydrocannabinol and closely related semi-synthetic cannabinoids: A comprehensive review. Drug Test. Anal. 2023, 10.1002/dta.3519. 37269160

[ref47] Graziano S.; Varì M. R.; Pichini S.; Busardo F. P.; Cassano T.; Di Trana A. Hexahydrocannabinol pharmacology, toxicology, and analysis: the first evidence for a recent new psychoactive substance. Curr. Neuropharmacol. 2023, 10.2174/1570159X21666230623104624. PMC10616920 37357519

[ref48] Russo F.; Vandelli M. A.; Biagini G.; et al. Synthesis and pharmacological activity of the epimers of hexahydrocannabinol (HHC). Sci. Rep. 2023, 13, 11061 10.1038/s41598-023-38188-5. 37422571 PMC10329643

