# Supplementary material for: Studies Pertaining to the Emerging Cannabinoid Hexahydrocannabinol (HHC)
Source: ACS Chem Biol. 2023 Aug 14;18(9):2023–9. doi: 10.1021/acschembio.3c00254 (PMC10510108; doi:10.1021/acschembio.3c00254)
Supplement: Supplementary file 1 — cb3c00254_si_001.pdf [file cb3c00254_si_001.pdf]

Studies pertaining to the emerging cannabinoid hexahydrocannabinol (HHC)

Daniel J. Nasrallah and Neil K. Garg*

Department of Chemistry & Biochemistry, University of California
Los Angeles, California 90095, United States
*neilgarg@chem.ucla.edu

Supporting Information – Table of Contents

Materials and Methods S2

Experimental Procedures S3
A. Optimized HAT Conditions to Access HHCs **4a** and **4b** from Δ^9-THC (**1**) S3
B. Optimization of Δ^9-THC (1) Reduction (Table 1) S3
C. Optimized HAT Conditions to Access HHCs **4a** and **4b** from Δ^8-THC (**3**) S7

Computational Investigation S8
A. Computational Methods S8
B. Cartesian Coordinates of Optimized Structures S8

CB_1 and CB_2 Receptor Studies Conducted by Eurofins Discovery S11
A. Human CB_1 Cannabinoid Receptor (Agonist Radioligand), Binding Assay S11
Table S1: Data for **4a** S11
Table S2: Data for **4b** S12
Table S3: Data for **1** S12
B. Human CB_2 Cannabinoid Receptor (Agonist Radioligand), Binding Assay S12
Table S4: Data for **4a** S13
Table S5: Data for **4b** S13
Table S6: Data for **1** S13
C. Human Cannabinoid CB_1 Receptor (Agonist Effect), GPCR Functional Assay S14
Table S7: Data for **4a** S14
Table S8: Data for **4b** S15
Table S9: Data for **1** S15
D. Human Cannabinoid CB_2 Receptor (Agonist Effect), GPCR Functional Assay S15
Table S10: Data for **4a** S16
Table S11: Data for **4b** S16
Table S12: Data for **1** S17

Examination of Certificate of Analyses of Commercial HHC Samples S18
Table S13: Ratios of **4a** to **4b** of Sixty-one Commercial Samples S18

^{1}H NMR Spectra S20

References S24

Materials and Methods. Unless stated otherwise, reactions were conducted in flame-dried glassware under an atmosphere of nitrogen using anhydrous solvents (passed through activated alumina columns or dried over 4Å molecular sieves). All commercially obtained reagents were used as received unless otherwise specified. Δ^9-THC (**1**) was purchased from Cayman Chemical (https://www.caymanchem.com; item #12068) under DEA approval.[1] Δ^8-THC (**3**) is a known compound and was prepared following the literature procedure.[2] The ^{1}H NMR spectral data matched that reported in the literature.[3] Diimide 5 is a known compound and was prepared following the literature procedure.[4] $Fe(acac)_3$ (99%), Pd/C (10% weight), and $[Ir(cod)PCy_3Py]PF_6$ (Crabtree's Catalyst) were purchased from Strem Chemicals. Absolute ethanol was obtained from EMD Millipore Corporation. Phenylsilane ($PhSiH_4$, 97%) and reagent grade *n*-propanol were obtained from Oakwood Chemicals. Thiophenol (PhSH, 97%), PtO_2 (Adams' catalyst), Pt/C (5% weight), Rh/C (5% weight), $RhCl(PPh)_3$ (Wilkinson's catalyst), $CoCl_3$, and $LiAlH_4$ (2.0 M solution in THF) were obtained from Sigma Aldrich. AcOH was obtained from Fisher Scientific. H_2 gas was obtained from Airgas Inc. Unless stated otherwise, reactions were performed at room temperature (approximately 23 °C). Thin layer chromatography (TLC) was conducted with EMD gel 60 F254 pre-coated plates (0.25 mm) and visualized using a combination of UV light and potassium permanganate or anisaldehyde staining. Preparative thin layer chromatography (pTLC) was conducted with EMD gel 60 F254 pre-coated plates (0.5 mm) and visualized using UV light and anisaldehyde staining. Silicycle Siliaflash P60 (particle size 0.040–0.063 mm) was used for flash column chromatography. ^{1}H NMR were recorded on Bruker spectrometers (400 MHz and 500 MHz) and are reported relative to deuterated solvent signals.

General note about HHC stereochemistry:

The language in the literature about the stereochemistry of HHCs isomers is convoluted. Specifically, regarding "HHC enantiomers." This is due to the fact that HHCs possess 3 stereocenters and therefore 8 stereoisomers. In our study, the C10a (*S*) and C6a (*R*) stereocenters remain fixed based on the stereochemistry of THC. The C9 stereocenter formed in HHC create the diastereomers discussed, referred to as either (*9R*) or (*9S*)-HHC (**4a** and **4b** respectively).

Experimental Procedures.

A. Optimized HAT Conditions to Access HHCs 4a and 4b from Δ[9]-THC (1):

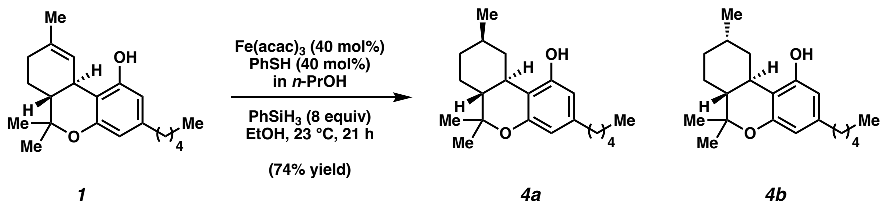

A solution of Δ[9]-THC (**1**, 0.292 mL of a 51.4 mg/mL solution in hexanes; 15.0 mg, 47.7 µmol, 1.00 equiv) in a 1-dram vial containing a magnetic stir bar was concentrated under reduced pressure to afford a light-yellow oil. The material was then dissolved in ethanol (477 µL dried over 4Å MS, 0.100 molar). Phenylsilane (23.7 µL, 191 µmol, 4.00 equiv) via a micro syringe, $Fe(acac)_3$ (3.37 mg, 9.54 µmol, 0.200 equiv) weighted out under air, and thiophenol (19.1 µL of a 0.500 molar solution in *n*-propanol, 9.54 µmol, 0.200 equiv) via a micro syringe were added. The reaction was then purged with nitrogen for 10 mins. Then, the reaction was allowed to stir at 23 °C under nitrogen for 17 h. After 17 h, a second portion of phenylsilane (23.7 µL, 191 µmol, 4.00 equiv), $Fe(acac)_3$ (3.37 mg, 9.54 µmol, 0.200 equiv), and thiophenol (19.1 µL of a 0.500 molar solution in *n*-propanol, 9.54 µmol, 0.200 equiv) were added sequentially. The reaction was purged with nitrogen for 10 mins. The reaction was left to stir at 23 °C under nitrogen for another 4 h. After a total reaction time of 21 h, the reaction was concentrated under reduced pressure and then filtered through a monster pipette containing silica (4.0 cm) with 40% EtOAc in hexane (10 mL) followed by benzene (2.0 mL). The crude material was purified by preparative TLC (100% benzene) to afford **4** as a clear oil (74% yield, 9.5:1 dr favoring **4a**, average of two experiments: 73% yield, 9.8:1 dr favoring **4a** and 75% yield, 9.1:1 dr favoring **4a**). Spectral data matched those reported in the literature.[5,6] Recovered starting material **1** (10% yield, average of two experiments: 9% yield and 10% yield) was also collected as a clear oil. To obtain an analytical sample for biological evaluation, the mixture of diastereomers was further purified by iterative preparative TLC (100% benzene) to afford **4a** with >19:1 dr. (*9R*)-HHC **4a**: ^{1}H NMR (500 MHz, $CDCl_3$): δ 6.25 (d, *J* = 1.5 Hz, 1H), 6.08 (d, *J* = 1.5 Hz, 1H), 4.64 (s, 1H), 3.06–2.99 (m, 1H), 2.50–2.35 (m, 3H), 1.91–1.80 (m, 2H), 1.67–1.58 (m, 1H), 1.58–1.53 (m, 3H), 1.45 (td, *J* = 11.4, 2.6 Hz, 1H), 1.36 (s, 3H), 1.33–1.27 (m, 3H), 1.17–1.07 (m, 2H), 1.07 (s, 3H), 0.94 (d, *J* = 6.7 Hz, 3H), 0.90–0.86 (m, 3H), 0.82–0.74 (m, 1H).; ^{13}C NMR (125 MHz, C_6D_6): δ 155.8, 155.5, 142.5, 110.7, 110.6, 107.8, 76.7, 49.4, 39.4, 36.0, 35.9, 35.8, 33.1, 31.8, 31.2, 28.3, 28.0, 23.0, 22.9, 19.2, 14.2; HRMS-ESI (*m/z*) $[M + H]^+$ calcd for $C_{21}H_{33}O_2^+$, 317.2480; found 317.2487.

B. Optimization of Δ[9]-THC (1) Reduction (Table 1):

Adams' catalyst conditions: (Table 1, entry 1):[7]

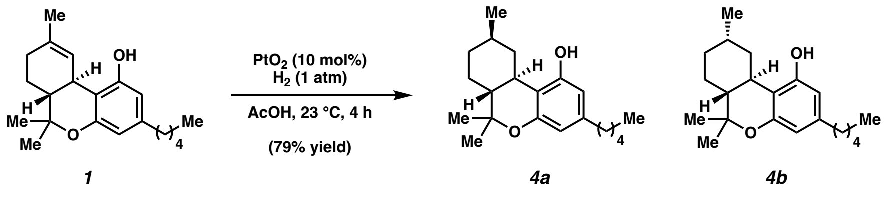

A solution of Δ[9]-THC (**1**, 0.230 mL of a 65.2 mg/mL solution in ethanol; 15.0 mg, 48.0 µmol, 1.00 equiv) was concentrated under reduced pressure to afford a light-yellow oil. Then, PtO_2 (1.1 mg, 4.8

μmol, 0.100 equiv) followed by acetic acid (2.00 mL, 35.0 mmol, 0.0240 molar) were added to the reaction vial. The reaction vial was first sparged with nitrogen for 1 minute and then hydrogen gas (1 atm) was bubbled through the reaction mixture for 10 mins. The reaction was left to stir under hydrogen gas for 4 h. After 4 h, the reaction was filtered through a plug of celite (4 cm) in a monster pipette eluting with EtOAc (10 mL). The organic layer was rinsed with water (2 mL x 2), then the organic layer was collected, dried over $MgSO_4$, and concentrated under reduced pressure. The crude material was purified by preparative TLC (100% benzene) to afford **4** as a clear oil (12.0 mg, 79% yield, 1.1:1 dr favoring **4a**).

Pt/C conditions: (Table 1, entry 2):

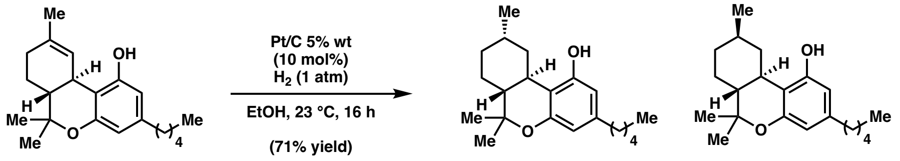

1 *4b* *4a*

To a solution of Δ^9-THC (**1**, 0.230 mL of a 65.2 mg/mL solution in ethanol; 15.0 mg, 48.0 μmol, 1.00 equiv) was added Pt/C (19.0 mg, 5% wt, 4.8 μmol, 0.100 equiv) and ethanol (1.77 mL dried over 4Å MS, 0.0270 molar). The reaction vial was first sparged with nitrogen for 1 minute and then hydrogen gas (1 atm) was bubbled through the reaction mixture while it stirred for 10 mins. The reaction was left to stir under hydrogen gas for 16 h. After 16 h, the reaction was filtered through a plug of celite (4 cm) in a monster pipette eluting with EtOAc (10 mL). The crude material was concentrated under reduced pressure and then purified via preparative TLC (100% benzene) to afford **4** as a clear oil (10.7 mg, 71% yield, 1:1.1 dr favoring **4b**).

Rh/C conditions: (Table 1, entry 3):

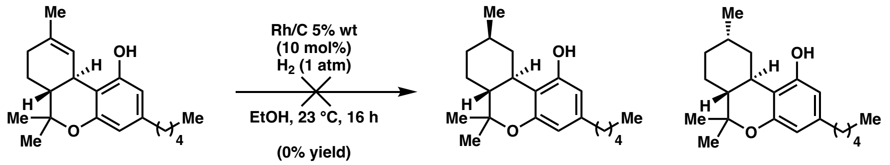

1 *4a* *4b*

To a solution of Δ^9-THC (**1**, 0.230 mL of a 65.2 mg/mL solution in ethanol; 15.0 mg, 48.0 μmol, 1.00 equiv) was added Rh/C (9.5 mg, 5% wt, 4.8 μmol, 0.100 equiv) and ethanol (1.77 mL dried over 4Å MS, 0.027 molar). The reaction vial was first sparged with nitrogen for 1 minute and then hydrogen gas (1 atm) was bubbled through the reaction mixture for 10 mins. The reaction was left to stir under hydrogen gas for 16 h. After 16 h, the reaction was filtered through a plug of celite (4 cm) in a monster pipette eluting with EtOAc (10 mL). The crude material was then concentrated under reduced pressure and purified via preparative TLC plate (100% benzene) to give recovered starting material **1** (12.7 mg, 85% yield) as a light-yellow oil.

Pd/C conditions: (Table 1, entry 4):[8]

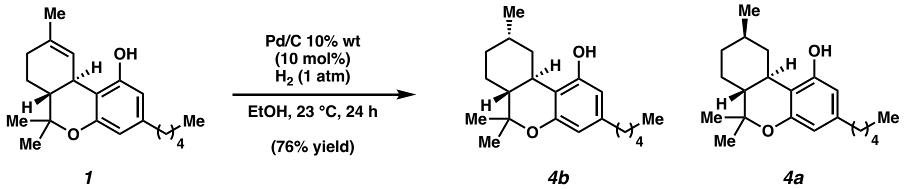

To a solution of Δ[9]-THC (**1**, 0.230 mL of a 65.2 mg/mL solution in ethanol; 15.0 mg, 48.0 µmol, 1.00 equiv) was added Pd/C (5.1 mg, 10% wt, 4.8 µmol, 0.100 equiv) and ethanol (1.77 mL dried over 4Å MS, 0.0270 molar). The reaction vial was first sparged with nitrogen for 1 minute and then hydrogen gas (1 atm) was bubbled through the reaction mixture for 10 mins. The reaction was left to stir under hydrogen gas for 24 h. After 24 h, the reaction was filtered through a plug of celite (4 cm) in a monster pipette eluting with EtOAc (10 mL). The crude material was then concentrated under reduced pressure and purified via preparative TLC plate (100% benzene) to give the desired product **4** (11.5 mg, 76% yield, 1:3.9 dr favoring **4b**) as a clear oil. To obtain an analytical sample for biological evaluation, the mixture of diastereomers was further purified by iterative preparative TLC (100% benzene) to afford **4b** with >19:1 dr. (*9S*)-HHC **4b**: ^{1}H NMR (500 MHz, $CDCl_3$): δ 6.24 (d, J = 1.5 Hz, 1H), 6.07 (d, J = 1.5 Hz, 1H), 4.63 (s, 1H), 2.87 (dtd, J = 13.2, 2.6, 1.4 Hz, 1H), 2.67 (td, J = 11.5, 3.0 Hz, 1H), 2.42 (td, J = 7.6, 2.9 Hz, 2H), 2.16–2.07 (m, 1H), 1.69–1.59 (m, 3H), 1.59–1.51 (m, 2H), 1.50–1.44 (m, 1H), 1.36 (s, 3H), 1.34–1.27 (m, 6H), 1.13 (d, J = 7.4 Hz, 3H), 1.09 (s, 3H), 0.91–0.85 (m, 3H).; ^{13}C NMR (125 MHz, C_6D_6): δ 156.1, 155.3, 142.4, 110.8, 110.7, 107.8, 76.6, 50.3, 36.5, 35.9, 32.5, 31.9, 31.2, 29.9, 28.4, 27.8, 23.3, 23.0, 19.2, 19.0, 14.2; HRMS-ESI (*m/z*) $[M + H]^+$ calcd for $C_{21}H_{33}O_2^+$, 317.2480; found 317.2482.

Crabtree's catalyst conditions: (Table 1, entry 5):[8]

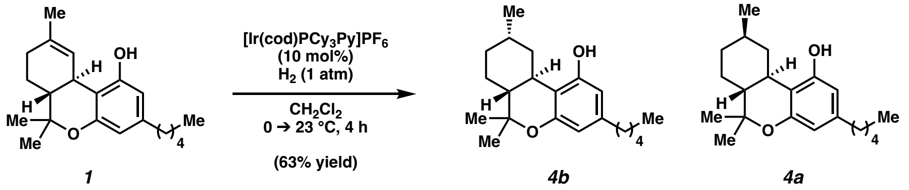

A solution of Δ[9]-THC (**1**, 0.206 mL of a 65.2 mg/mL solution in ethanol; 13.4 mg, 42.6 µmol, 1.00 equiv) was concentrated under reduced pressure to afford a light-yellow oil. Then, Crabtree's catalyst (3.43 mg, 4.26 µmol, 0.100 equiv) was added inside the glove box. Next, CH_2Cl_2 (1.78 mL, 42.6 mmol, 0.0240 molar) was then added outside of the glovebox and the vial was cooled to 0 °C in an ice bath. The reaction vial was first sparged with nitrogen for 1 minute and then hydrogen gas (1 atm) was bubbled through the reaction mixture for 10 mins. The reaction was left to stir under hydrogen gas for 4 h and warm to 23 °C. After 4 h, the reaction was filtered through a plug of celite (4 cm) in a monster pipette eluting with EtOAc (10 mL). The crude material was then concentrated under reduced pressure and purified via preparative TLC plate (100% benzene) to give the desired product **4** (8.50 mg, 63% yield, 1:2.0 dr favoring **4b**) as a clear oil as well as recovered starting material **1** (1.9 mg, 14% yield) as a light-yellow oil.

Wilkinson's catalyst conditions: (Table 1, entry 6):[9]

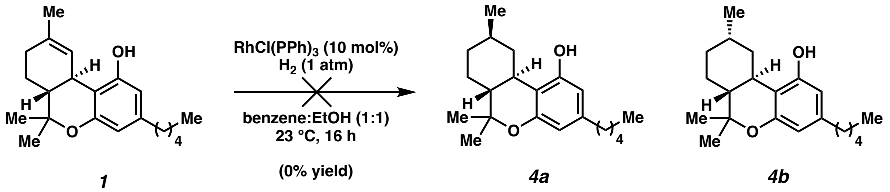

Wilkinson's catalyst (4.4 mg, 4.8 µmol, 0.100 equiv) was weighed out and added to a vial. Next, a solution of Δ[9]-THC (**1**, 0.230 mL of a 65.2 mg/mL solution in ethanol; 15.0 mg, 48.0 µmol, 1.00 equiv), ethanol (0.250 mL dried over 4Å MS, 0.100 molar), and benzene (0.250 mL, 0.100 molar) were added. The reaction vial was first sparged with nitrogen for 1 minute and then hydrogen gas (1 atm) was bubbled through the reaction mixture for 10 mins. The reaction was left to stir under hydrogen gas for 16 h. After 16 h, the reaction was filtered through a plug of celite (4 cm) in a monster pipette eluting with EtOAc (10 mL). The crude material was then concentrated under reduced pressure and purified via preparative TLC plate (100% benzene) to give recovered starting material **1** (14.0 mg, 93% yield) as a light-yellow oil.

Diimide **5** conditions: (Table 1, entry 7):[10]

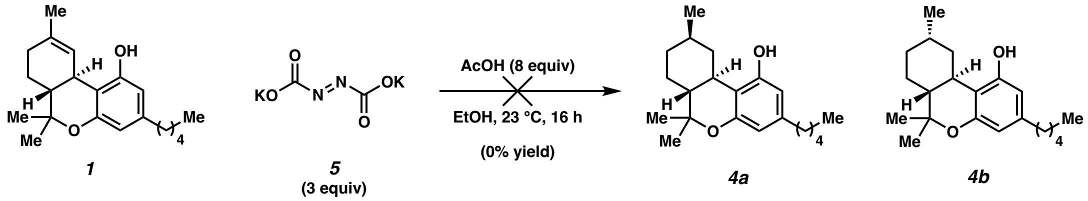

Diimide **5** (27.8 mg, 143.0 µmol, 3.00 equiv) was added to a vial. Next, a solution of Δ[9]-THC (**1**, 0.230 mL of a 65.2 mg/mL solution in ethanol; 15.0 mg, 48.0 µmol, 1.00 equiv), ethanol (0.77 mL dried over 4Å MS, 0.062 molar), and acetic acid (21.8 µL, 382 µmol, 8.00 equiv) were added. The reaction was left to stir for 16 h at 23 °C under nitrogen. After 16 h, the reaction was filtered through a plug of celite (4 cm) in a monster pipette eluting with EtOAc (10 mL). The crude material was then concentrated under reduced pressure and purified via a monster pipette silica column (4 cm) eluting with benzene (10 mL) to give recovered starting material **1** (12.5 mg, 83% yield) as a light-yellow oil.

$CoCl_2$/$LiAlH_4$ conditions: (Table 1, entry 8):[11]

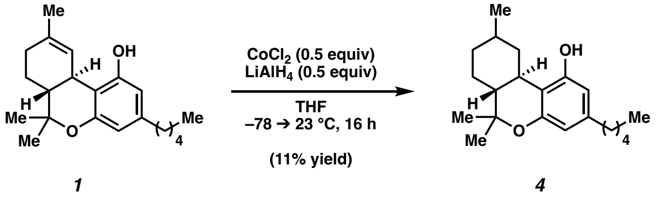

A solution of Δ[9]-THC (**1**, 0.230 mL of a 65.2 mg/mL solution in ethanol; 15.0 mg, 48.0 µmol, 1.00 equiv) was concentrated under reduced pressure to afford a light-yellow oil. Then, $CoCl_2$ (3.10 mg, 24.0 µmol, 0.500 equiv) and THF (2.00 mL, 48.0 mmol, 0.0240 molar) were added to the vial inside a glove box. The vial was removed from the glove box and cooled to –78 °C in a dry ice acetone bath under nitrogen. Then, $LiAlH_4$ (12.0 µL of a 2.0 molar solution in THF, 24.0 µmol, 0.500 equiv) was added

slowly via a micro syringe. The reaction was left to stir and warm to 23 °C for 16 h. After 16 h, the reaction was filtered through a plug of celite (4 cm) in a monster pipette eluting with EtOAc (10 mL). The crude material was then concentrated under reduced pressure and purified via preparative TLC plate (100% benzene) to give the desired product **4** (1.60 mg, 11% yield, dr could not be determined) as a clear oil as well as recovered starting material **1** (10.2 mg, 68% yield) as a light-yellow oil.

West HAT conditions: (Table 1, entry 9):[12]

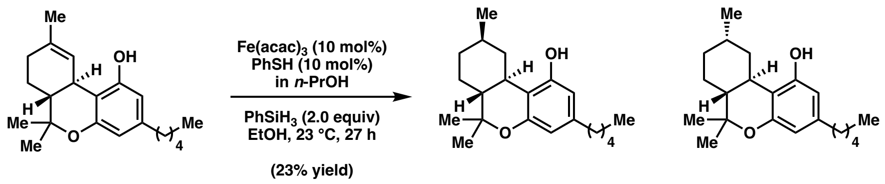

1 *4a* *4b*

A solution of Δ^9-THC (**1**, 0.30 mL of a 50.1 mg/mL solution in hexanes; 15.0 mg, 47.7 μmol, 1.00 equiv) was added to a vial. Ethanol (477 μL dried over 4Å MS, 0.100 molar) was then added to the vial. Phenylsilane (5.20 μL, 42.0 μmol, 2.00 equiv) via a micro syringe, $Fe(acac)_3$ (0.740 mg, 2.10 μmol, 0.200 equiv) weighted out under air, and thiophenol (4.20 μL of a 0.500 molar solution in n-propanol, 2.10 μmol, 0.200 equiv) via a micro syringe were added. The reaction was then purged with nitrogen for 10 mins and then the nitrogen line removed. The reaction was left to stir for 27 h. After 27 h, the reaction was filtered through a plug of silica (4 cm) in a monster pipette eluting with 40% EtOAc in hexanes (10 mL) and benzene (2 mL). The crude material was then concentrated under reduced pressure and purified via preparative TLC plate (100% benzene) to give the desired product **4** (23% yield, average of two experiments: 10% yield, 9.2:1 dr favoring **4a,** and 36% yield, 9.6:1 dr favoring **4a**) as a clear oil as well as recovered starting material **1** (38% yield, average of two experiments: 37% yield and 39% yield) as a light-yellow oil.

C. Optimized HAT Conditions to Access HHCs 4a and 4b from Δ^8-THC (3):

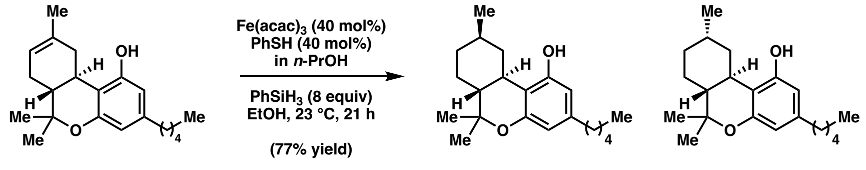

3 *4a* *4b*

A solution of Δ^8-THC (**3**, 0.303 mL of a 49.6 mg/mL solution in ethanol; 15.0 mg, 47.7 μmol, 1.00 equiv) in a 1-dram vial containing a magnetic stir bar was concentrated under reduced pressure to afford a light-yellow oil. The material was then dissolved in ethanol (477 μL dried over 4Å MS, 0.100 molar). Phenylsilane (23.7 μL, 191 μmol, 4.00 equiv) via a microsyringe, $Fe(acac)_3$ (3.37 mg, 19.54 μmol, 0.200 equiv) weighted out in air, and thiophenol (19.7 μL of a 0.500 molar solution in n-propanol, 9.54 μmol, 0.200 equiv) via a micro syringe were added to the vial. The reaction was then purged with nitrogen for 10 mins. Then the reaction was allowed to stir at 23 °C under nitrogen for 17 h. After 17 h, a second portion of phenylsilane (23.7 μL, 191 μmol, 4.00 equiv), $Fe(acac)_3$ (3.37 mg, 19.54 μmol, 0.200 equiv), and thiophenol (19.7 μL of a 0.500 molar solution in n-propanol, 9.54 μmol, 0.200 equiv) were added sequentially. The reaction was left to stir at 23 °C under nitrogen for another 4 h. After a total reaction time of 21 h, the reaction was concentrated under reduced pressure and then filtered through a monster pipette containing silica (4.0 cm) with 40% EtOAc in hexane (10 mL) followed by benzene (2.0 mL). The crude material was purified by preparative TLC (100% benzene) to afford the desired product **4** as

a clear oil (77% yield, 11.0:1 dr favoring **4a,** average of two experiments: 76% yield, 11.3:1 dr favoring **4a** and 78% yield, 10.6:1 dr favoring **4a**). Spectral data matched those reported in the literature.[5,6]

Computational Investigation.

A. Computational Methods:

All calculations were carried out with Spartan 20.[13] An initial geometry optimization was performed with ωB97X-D functional[14] and the 6-31G* basis set. The resultant structures were then submitted for a conformational search using molecular mechanics. Four rounds of bond rotations were investigated for the hydroxyl group as well as chain flips of all C(sp^3) atoms in the rings. The conformers obtained were then optimized using the same level of theory as for geometry optimizations. Frequency analysis was conducted to verify the stationary points to be minima. Optimized structures are presented using CYLview.[15]

B. Cartesian Coordinates of Optimized Structures:

4a-Me

C	0.391467	-2.079562	-1.232574
C	2.451928	-1.974637	0.609754
C	0.394824	-1.019473	-0.313119
C	1.411942	-3.024641	-1.272326
C	2.464312	-2.968940	-0.356479
C	1.419411	-1.033505	0.641855
H	3.225851	-1.913169	1.368177
H	1.382301	-3.818485	-2.017261
O	1.483077	-0.138909	1.662169
C	0.277211	0.571433	2.009109
C	-0.286524	1.165736	0.711639
C	-0.660300	0.068471	-0.300902
C	-0.906573	0.746412	-1.661642
C	-2.048379	1.768245	-1.574101
C	-1.784381	2.798405	-0.470510
C	-1.452853	2.143681	0.873790
H	-0.937426	3.431685	-0.774046
H	-2.650456	3.463417	-0.364150
H	-1.209368	2.920607	1.606672
H	-2.336104	1.610992	1.252860
O	-0.668074	-2.164188	-2.090382
H	-0.541819	-2.933366	-2.658361
C	3.556853	-4.007803	-0.385117
H	3.772750	-4.330968	-1.408419
H	3.266137	-4.896925	0.187242
C	-0.681893	-0.390055	2.714968
H	-0.938452	-1.242075	2.079439
H	-1.607745	0.124033	2.992494
H	-0.214611	-0.775045	3.626372
C	0.757696	1.648716	2.975152

H	1.349587	1.186176	3.770364
H	1.386473	2.380591	2.458267
H	-0.086613	2.170380	3.434392
C	-2.285436	2.447620	-2.921926
H	-3.124573	3.150802	-2.871262
H	-1.396109	3.009512	-3.233685
H	-2.506525	1.711617	-3.702723
H	-1.143530	0.000798	-2.422226
H	0.017549	1.251002	-1.980624
H	-2.961366	1.215190	-1.303486
H	-1.616715	-0.381228	0.010623
H	0.553890	1.729797	0.277802
H	4.482565	-3.622345	0.052181

Imaginary frequencies: 0

4b-Me

C	-0.168084	-2.349485	-0.636575
C	2.002963	-2.242760	1.075040
C	0.200928	-1.096819	-0.122878
C	0.555185	-3.503984	-0.353619
C	1.661960	-3.455739	0.496501
C	1.266169	-1.090778	0.786425
H	2.829187	-2.161648	1.774177
H	0.247445	-4.453023	-0.790366
O	1.655992	0.027509	1.452325
C	0.718533	1.119185	1.538693
C	0.217989	1.398774	0.115259
C	-0.525436	0.186627	-0.473283
C	-0.702310	0.433261	-1.984680
C	-1.507158	1.714195	-2.270235
C	-0.880533	2.921224	-1.553597
C	-0.623338	2.665121	-0.063571
H	0.080942	3.155068	-2.030806
H	-1.517268	3.805657	-1.679442
H	-1.574655	2.562606	0.475538
H	-0.111233	3.533608	0.364771
O	-1.285008	-2.403266	-1.421022
H	-1.421647	-3.317213	-1.697105
C	2.428269	-4.712400	0.824385
H	1.947325	-5.260543	1.643388
H	2.477505	-5.385051	-0.037876
C	-0.393844	0.746536	2.521492
H	-0.921869	-0.158239	2.208619
H	-1.122204	1.559652	2.604297
H	0.033893	0.564402	3.512014
C	1.552591	2.269670	2.091417
H	2.071157	1.941061	2.996790
H	2.302607	2.590302	1.361448

H	0.921950	3.125510	2.347992
C	-2.993188	1.551279	-1.929485
H	-3.161830	1.401608	-0.857643
H	-3.419356	0.687314	-2.450872
H	-3.558590	2.441215	-2.228545
H	-1.194060	-0.419231	-2.455734
H	0.295713	0.519538	-2.436587
H	-1.445989	1.901584	-3.350608
H	-1.528340	0.139944	-0.023603
H	1.136185	1.531291	-0.478724
H	3.451449	-4.483560	1.136288

Imaginary frequencies: 0

CB_1 and CB_2 Receptor Studies Conducted by Eurofins Discovery.

A. Human CB_1 Cannabinoid Receptor (Agonist Radioligand), Binding Assay:[16]

Evaluation of the affinity of compounds for the human CB_1 cannabinoid receptor in transfected Chem-1 cells determined in a radioligand binding assay.

Experimental protocol: Cell membrane homogenates (20 µg protein) are incubated for 30 min at 22 °C with 2 nM [^{3}H]CP 55940 in the absence or presence of the test compound in a buffer containing 50 mM Tris-HCl (pH 7.4), 5 mM $MgCl_2$, 2.5 mM EDTA and 0.3% BSA. Nonspecific binding is determined in the presence of 10 µM WIN 55212-2. Following incubation, the samples are filtered rapidly under vacuum through glass fiber filters (GF/B, Packard) presoaked with 0.3% PEI and rinsed several times with an ice-cold buffer containing 50 mM Tris-HCl (pH 7.4), 500 mM NaCl and 0.1% BSA using a 96-sample cell harvester (Unifilter, Packard). The filters are dried, and then counted for radioactivity in a scintillation counter (Topcount, Packard) using a scintillation cocktail (Microscint 0, Packard). The results are expressed as a percent inhibition of the control radioligand specific binding. The standard reference compound is CP 55940 which is tested in each experiment at several concentrations to obtain a competition curve from which its IC_{50} is calculated.

The results are expressed as a percent inhibition of control specific binding: 100–(measured specific binding/control specific binding*100) obtained in the presence of the test compounds.

The IC_{50} values (concentration causing a half-maximal inhibition of control specific binding) and Hill coefficients (nH) were determined by non-linear regression analysis of the competition curves generated with mean replicate values using Prism equation curve fitting, log(inhibitor) vs response - variable slope (four parameter).[17] The bottom of the line was constrained to zero unless otherwise noted. The inhibition constants (K_i) were calculated using the Cheng Prusoff equation using Prism software.

Table S1: Data for 4a

	% inhibition of control specific binding		Top: 111
Log[M]	1st	2nd	Bottom: 0
–8.34	16.40	–23.40	nH: 1.1
–7.86	27.40	29.00	$IC_{50} = 4.71*10^{-8}$ M
–7.39	50.10	52.90	K_i = 15 nM
–6.91	82.50	83.30	
–6.43	95.20	100.00	
–5.95	105.50	107.60	
–5.48	112.10	113.60	
–5.00	113.00	110.40	

Table S2: Data for 4b

	% inhibition of control specific binding		Top: 106
Log[M]	1st	2nd	Bottom: –9
–8.34	–10.50	–7.70	nH: 1.1
–7.86	–9.00	–6.10	$IC_{50} = 5.67*10^{-7}$ M
–7.39	–4.50	0.20	K_i = 180 nM
–6.91	8.70	5.50	
–6.43	35.70	35.70	
–5.95	66.50	69.40	
–5.48	88.80	95.80	
–5.00	100.40	101.20	

Table S3: Data for 1

	% inhibition of control specific binding		Top: 113
Log[M]	1st	2nd	Bottom: 0
–8.34	11.00	11.70	nH: 0.8
–7.86	31.50	29.10	$IC_{50} = 4.61*10^{-8}$ M
–7.39	62.50	57.30	K_i = 14 nM
–6.91	57.70	90.80	
–6.43	99.10	86.90	
–5.95	109.40	106.90	
–5.48	111.20	109.50	
–5.00	110.50	112.00	

B. Human CB_2 Cannabinoid Receptor (Agonist Radioligand), Binding Assay:[18]

Evaluation of the affinity of compounds for the human CB_2 cannabinoid receptor in transfected CHO cells determined in a radioligand binding assay.

Experimental protocol: Cell membrane homogenates (12 µg protein) are incubated for 120 min at 37 °C with 0.8 nM [^{3}H]WIN 55212-2 in the absence or presence of the test compound in a buffer containing 50 mM HEPES/Tris (pH 7.4), 5 mM $MgCl_2$, 2.5 mM EGTA and 0.1% BSA. Nonspecific binding is determined in the presence of 5 µM WIN 55212-2. Following incubation, the samples are filtered rapidly under vacuum through glass fiber filters (GF/B, Packard) presoaked with 0.3% PEI and rinsed several times with ice-cold 50 mM Tris-HCl using a 96-sample cell harvester (Unifilter, Packard). The filters are dried, and then counted for radioactivity in a scintillation counter (Topcount, Packard) using a scintillation cocktail (Microscint 0, Packard). The results are expressed as a percent inhibition of the control radioligand specific binding. The standard reference compound WIN 55212-2 is tested in each experiment at several concentrations to obtain a competition curve from which its IC_{50} is calculated.

The results are expressed as a percent inhibition of control specific binding: 100–(measured specific binding/control specific binding*100) obtained in the presence of the test compounds.

The IC_{50} values (concentration causing a half-maximal inhibition of control specific binding) and Hill coefficients (nH) were determined by non-linear regression analysis of the competition curves generated

with mean replicate values using Prism equation curve fitting, log(inhibitor) vs response - variable slope (four parameter).[17] The bottom of the line was constrained to zero unless otherwise noted. The inhibition constants (K_i) were calculated using the Cheng Prusoff equation using Prism software.

Table S4: Data for 4a

	% inhibition of control specific binding		Top: 99
Log[M]	1st	2nd	Bottom: 0
–8.34	12.30	16.30	nH: 1.1
–7.86	47.90	41.40	$IC_{50} = 1.94*10^{-8}$ M
–7.39	64.90	67.50	K_i = 13 nM
–6.91	86.90	84.70	
–6.43	96.90	93.60	
–5.95	98.40	98.70	
–5.48	98.00	97.20	
–5.00	99.40	99.30	

Table S5: Data for 4b

	% inhibition of control specific binding		Top: 102
Log[M]	1st	2nd	Bottom: –11
–8.34	–9.40	2.60	nH: 0.7
–7.86	4.40	6.70	$IC_{50} = 1.63*10^{-7}$ M
–7.39	15.80	16.00	K_i = 110 nM
–6.91	41.60	41.40	
–6.43	62.60	61.40	
–5.95	79.40	78.70	
–5.48	88.30	88.00	
–5.00	100.00	96.20	

Table S6: Data for 1

	% inhibition of control specific binding		Top: 100
Log[M]	1st	2nd	Bottom: 0.3
–8.34	28.80	20.90	nH: 1.0
–7.86	50.30	51.20	$IC_{50} = 1.42*10^{-8}$ M
–7.39	74.30	67.60	K_i = 9.2 nM
–6.91	88.60	89.90	
–6.43	98.10	98.20	
–5.95	99.10	97.40	
–5.48	97.00	96.20	
–5.00	98.20	101.70	

C. Human Cannabinoid CB_1 Receptor (Agonist Effect), GPCR Functional Assay:[19]

Evaluation of the agonist activity of compounds at the human CB_1 receptor expressed in transfected CHO cells, determined by measuring their effects on cAMP modulation using the HTRF detection method.

Experimental protocol: The cells are suspended in HBSS buffer (Invitrogen) complemented with 20 mM HEPES (pH 7.4), then distributed in microplates at a density of 5.10^3 cells/well in the presence of either of the following: HBSS (basal control), the reference agonist at 30 nM (stimulated control) or various concentrations (EC_{50} determination), or the test compounds. Thereafter, the adenylyl cyclase activator forskolin is added at a final concentration of 25 µM. Following 30 min incubation at 37 °C, the cells are lysed and the fluorescence acceptor (D2-labeled cAMP) and fluorescence donor (anti-cAMP antibody labeled with europium cryptate) are added. After 60 min at room temperature, the fluorescence transfer is measured at λex = 337 nm and λem = 620 and 665 nm using a microplate reader (Envison, Perkin Elmer). The cAMP concentration is determined by dividing the signal measured at 665 nm by that measured at 620 nm (ratio). The results are expressed as a percent of the control response to 10 nM CP 55940. The standard reference agonist is CP 55940, which is tested in each experiment at several concentrations to generate a concentration-response curve from which its EC_{50} value is calculated.

The results are expressed as a percent of control agonist response: measured response/control response*100 obtained in the presence of the test compounds.

The EC_{50} values (concentration producing a half-maximal response) were determined by non-linear regression analysis of the concentration-response curves generated with mean replicate values using Prism equation curve fitting, log(agonist) vs response - variable slope (four parameter).[17] The bottom of the line was constrained to zero.

Table S7: Data for 4a

	% of control agonist response		Top: 98
Log[M]	1st	2nd	Bottom: 0
–10.25	–3.80	–19.80	$EC_{50} = 3.43*10^{-9}$ M
–9.77	–4.20	–15.80	
–9.29	–6.90	8.60	
–8.82	–3.10	24.80	
–8.34	52.20	81.30	
–7.86	94.30	89.90	
–7.39	100.00	97.60	
–6.91	98.00	88.20	
–6.43	97.80	98.00	
–5.95	101.50	91.60	
–5.48	97.10	87.60	
–5.00	111.50	114.60	

Table S8: Data for 4b

	% of control agonist response		Top: 99
Log[M]	1st	2nd	Bottom: 0
–8.34	–31.70	–16.50	$EC_{50} = 5.55*10^{-8}$ M
–7.86	12.50	1.40	
–7.39	30.40	45.60	
–6.91	75.90	86.00	
–6.43	86.60	86.80	
–5.95	98.40	91.80	
–5.48	98.60	97.30	
–5.00	115.00	107.60	

Table S9: Data for 1

	% of control agonist response		Top: 98
Log[M]	1st	2nd	Bottom: 0
–10.25	5.30	–7.50	$EC_{50} = 4.21*10^{-9}$ M
–9.77	3.40	–5.90	
–9.29	7.20	–8.30	
–8.82	-8.10	2.70	
–8.34	67.70	70.70	
–7.86	88.60	88.80	
–7.39	86.80	92.00	
–6.91	93.30	96.80	
–6.43	98.80	101.50	
–5.95	100.80	95.10	
–5.48	98.60	98.00	
–5.00	110.70	116.90	

D. Human Cannabinoid CB_2 Receptor (Agonist Effect), GPCR Functional Assay:[19]

Evaluation of the agonist activity of compounds at the human CB_2 receptor expressed in transfected CHO cells, determined by measuring their effects on cAMP modulation using the HTRF detection method.

Experimental protocol: The cells are suspended in HBSS buffer (Invitrogen) complemented with 20 mM HEPES (pH 7.4), then distributed in microplates at a density of $7.5x10^3$ cells/well in the presence of either of the following: HBSS (basal control), the reference agonist at 100 nM (stimulated control) or various concentrations (EC_{50} determination), or the test compounds. Thereafter, the adenylyl cyclase activator NKH 477 is added at a final concentration of 3 μM. Following 10 min incubation at 37 °C, the cells are lysed and the fluorescence acceptor (D2-labeled cAMP) and fluorescence donor (anti-cAMP antibody labeled with europium cryptate) are added. After 60 min at room temperature, the fluorescence transfer is measured at λex = 337 nm and λem = 620 and 665 nm using a microplate reader (Envison, Perkin Elmer). The cAMP concentration is determined by dividing the signal measured at 665 nm by that measured at 620 nm (ratio). The results are expressed as a percent of the control response to 100 nM

WIN 55212-2. The standard reference agonist is WIN 55212-2, which is tested in each experiment at several concentrations to generate a concentration-response curve from which its EC_{50} value is calculated.

The results are expressed as a percent of control agonist response: measured response/control response*100 obtained in the presence of the test compounds.

The EC_{50} values (concentration producing a half-maximal response) were determined by non-linear regression analysis of the concentration-response curves generated with mean replicate values using Prism equation curve fitting, log(agonist) vs response - variable slope (four parameter).[17] The bottom of the line was constrained to zero.

Table S10: Data for 4a

	% of control agonist response		Top: 70
Log[M]	1st	2nd	Bottom: 0
–10.25	21.20	1.00	$EC_{50} = 6.34*10^{-9}$ M
–9.77	11.50	10.60	
–9.29	10.00	13.00	
–8.82	-2.20	17.90	
–8.34	39.80	25.30	
–7.86	50.40	40.10	
–7.39	58.20	60.80	
–6.91	58.70	64.30	
–6.43	76.00	74.40	
–5.95	54.20	62.90	
–5.48	73.10	71.10	

Table S11: Data for 4b

	% of control agonist response		Top: 68
Log[M]	1st	2nd	Bottom: 0
–8.34	3.00	1.60	$EC_{50} = 5.38*10^{-8}$ M
–7.86	16.80	28.70	
–7.39	26.90	27.40	
–6.91	49.30	39.90	
–6.43	57.40	57.10	
–5.95	63.10	63.40	
–5.48	64.50	65.50	

Table S12: Data for 1

	% of control agonist response		Top: 63
Log[M]	1st	2nd	Bottom: 0
–10.25	4.50	1.00	EC_{50} = 2.47*10^{-9} M
–9.77	5.10	–8.20	
–9.29	0.40	11.30	
–8.82	26.20	30.30	
–8.34	32.60	45.80	
–7.86	56.60	52.60	
–7.39	56.70	62.20	
–6.91	56.60	58.90	
–6.43	60.00	67.60	
–5.95	67.60	66.50	
–5.48	59.10	64.10	

Examination of Certificate of Analyses of Commercial HHC Samples.

Table S13: Ratios of 4a to 4b of Sixty-one Commercial Samples, N/A = Not Applicable

Source*	Sample #	4a	4b	Ratio 4a to 4b (Given relative to 1)
A	1	62.74	36.13	1.7
A	2	63.64	35.17	1.8
A	3	62.77	35.4	1.8
B	1	48.2	24.7	2.0
B	2	53.5	25.9	2.1
B	3	61.9	30.7	2.0
B	4	69.1	28.6	2.4
B	5	52.3	22.9	2.3
C	1	N/A	N/A	N/A
C	2	N/A	N/A	N/A
C	3	N/A	N/A	N/A
C	4	N/A	N/A	N/A
C	5	45.11	21.41	2.1
C	6	N/A	N/A	N/A
C	7	48.98	23.3	2.1
C	8	N/A	N/A	N/A
C	9	N/A	N/A	N/A
C	10	53.09	25.3	2.1
C	11	N/A	N/A	N/A
C	12	N/A	N/A	N/A
C	13	53.09	25.3	2.1
D	1	60.84	30.83	2.0
D	2	N/A	N/A	N/A
D	3	60.32	30.5	2.0
D	4	N/A	N/A	N/A
D	5	N/A	N/A	N/A
E	1	56.51	33.4	1.7
E	2	46.06	44.93	1.0
E	3	55.66	39.88	1.4
E	4	52.68	44.7	1.2
F	1	66.5	30.5	2.2
F	2	46.5	53.4	0.9
F	3	49.47	43.74	1.1
F	4	38.44	53.55	0.7
F	5	49.3	42.7	1.2
F	6	46.77	52.3	0.9
F	7	48.95	43.31	1.1

F	8	43.06	48.6	0.9
F	9	47.3	42.8	1.1
F	10	48.3	41.8	1.2
F	11	33.19	59.79	0.6
F	12	46.3	41.6	1.1
F	13	46.4	40.4	1.1
F	14	49	43.36	1.1
F	15	42.45	47.59	0.9
G	1	61.72	29.02	2.1
G	2	62.64	29.22	2.1
G	3	41.9	53.6	0.8
H	1	44.97	52.13	0.9
H	2	48.18	55.63	0.9
H	3	44.61	51.83	0.9
H	4	46.38	53.08	0.9
H	5	N/A	N/A	N/A
H	6	N/A	N/A	N/A
I	1	0.18	0.37	0.5
I	2	0.4	0.46	0.9
I	3	58.6	36.3	1.6
I	4	56.5	37.7	1.5
I	5	11.32	45.72	0.2
I	6	N/A	N/A	N/A
I	7	35.48	52.52	0.7

* Sources "A" through "I" refer to different vendors who supply HHC products and provide Certificates of Analyses online for their products.

^{1}H NMR Spectra:

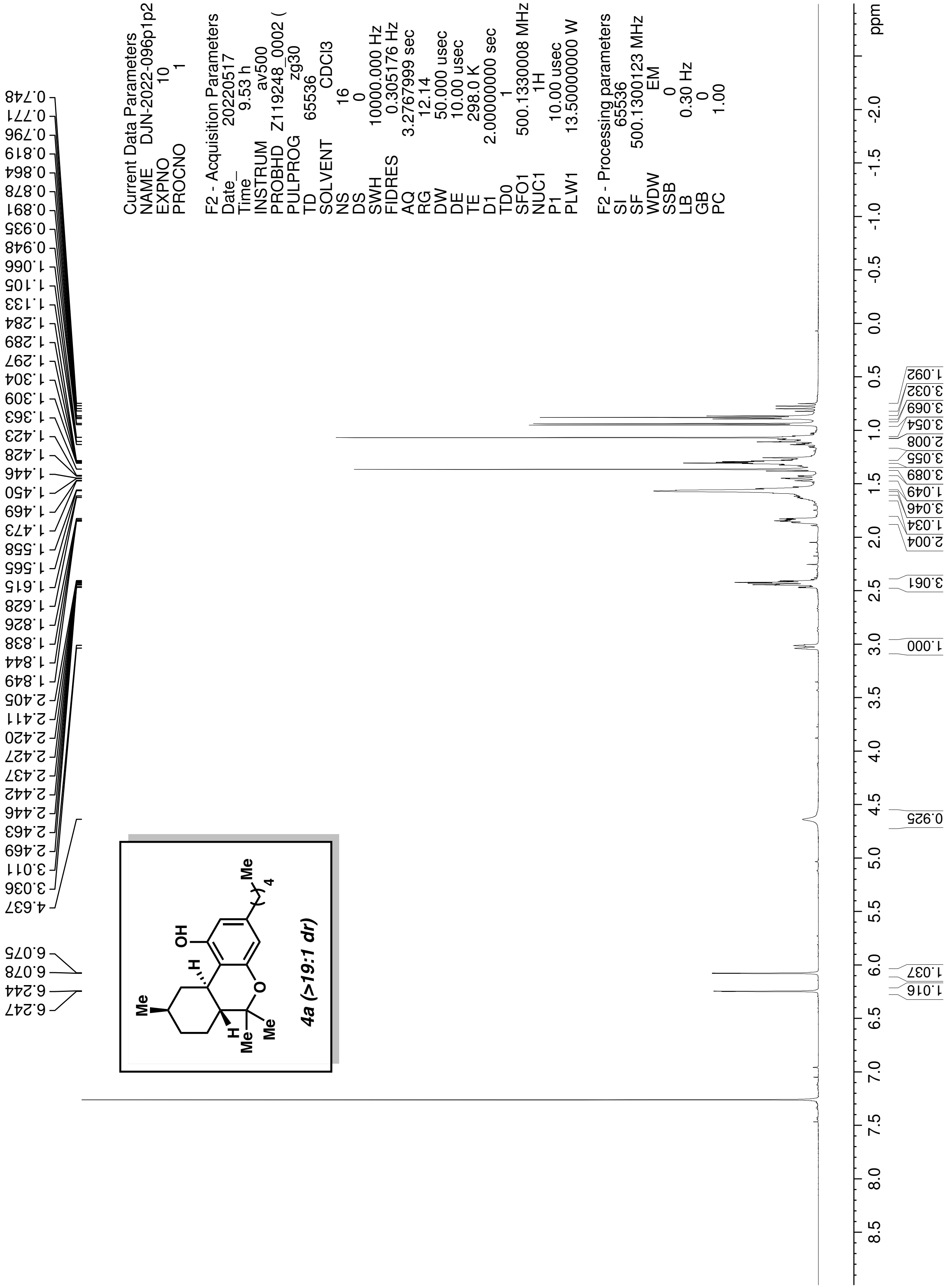
Current Data Parameters
NAME DJN-2022-096p1p2
EXPNO 10
PROCNO 1
F2 - Acquisition Parameters
Date_ 20220517
Time 9.53 h
INSTRUM av500
PROBHD Z119248_0002 (
PULPROG zg30
TD 65536
SOLVENT CDCl3
NS 16
DS 0
SWH 10000.000 Hz
FIDRES 0.305176 Hz
AQ 3.2767999 sec
RG 12.14
DW 50.000 usec
DE 10.00 usec
TE 298.0 K
D1 2.00000000 sec
TD0 1
SFO1 500.1330008 MHz
NUC1 1H
P1 10.00 usec
PLW1 13.50000000 W
F2 - Processing parameters
SI 65536
SF 500.1300123 MHz
WDW EM
SSB 0
LB 0.30 Hz
GB 0
PC 1.00
4a (>19:1 dr)
ppm

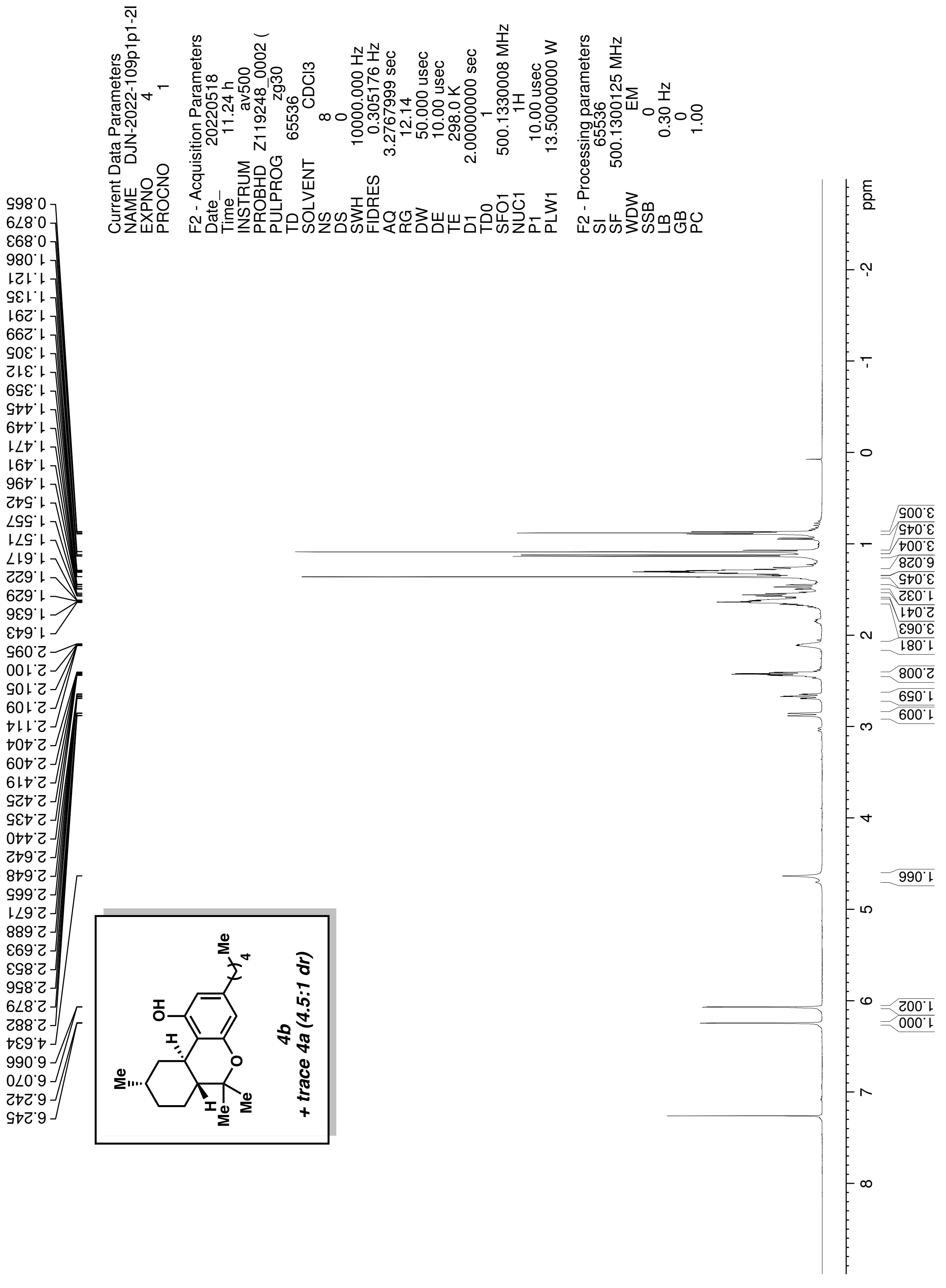
Current Data Parameters
NAME DJN-2022-109p1p1-2I
EXPNO 4
PROCNO 1
F2 - Acquisition Parameters
Date_ 20220518
Time_ 11.24 h
INSTRUM av500
PROBHD Z119248_0002 (
PULPROG zg30
TD 65536
SOLVENT CDCl3
NS 8
DS 0
SWH 10000.000 Hz
FIDRES 0.305176 Hz
AQ 3.2767999 sec
RG 12.14
DW 50.000 usec
DE 10.00 usec
TE 298.0 K
D1 2.00000000 sec
TD0 1
SFO1 500.1330008 MHz
NUC1 1H
P1 10.00 usec
PLW1 13.50000000 W
F2 - Processing parameters
SI 65536
SF 500.1300125 MHz
WDW EM
SSB 0
LB 0.30 Hz
GB 0
PC 1.00
0.865 0.879 0.893 1.086 1.121 1.135 1.291 1.299 1.305 1.312 1.359 1.445 1.449 1.471 1.491 1.496 1.542 1.557 1.571 1.617 1.622 1.629 1.636 1.643 2.095 2.100 2.105 2.109 2.114 2.404 2.409 2.419 2.425 2.435 2.440 2.642 2.648 2.665 2.671 2.688 2.693 2.853 2.856 2.879 2.882 4.634 6.066 6.070 6.242 6.245
3.005 3.045 3.004 6.028 3.045 1.032 2.041 3.063 1.081 2.008 1.059 1.009 1.066 1.002 1.000
ppm
4b
+ trace 4a (4.5:1 dr)

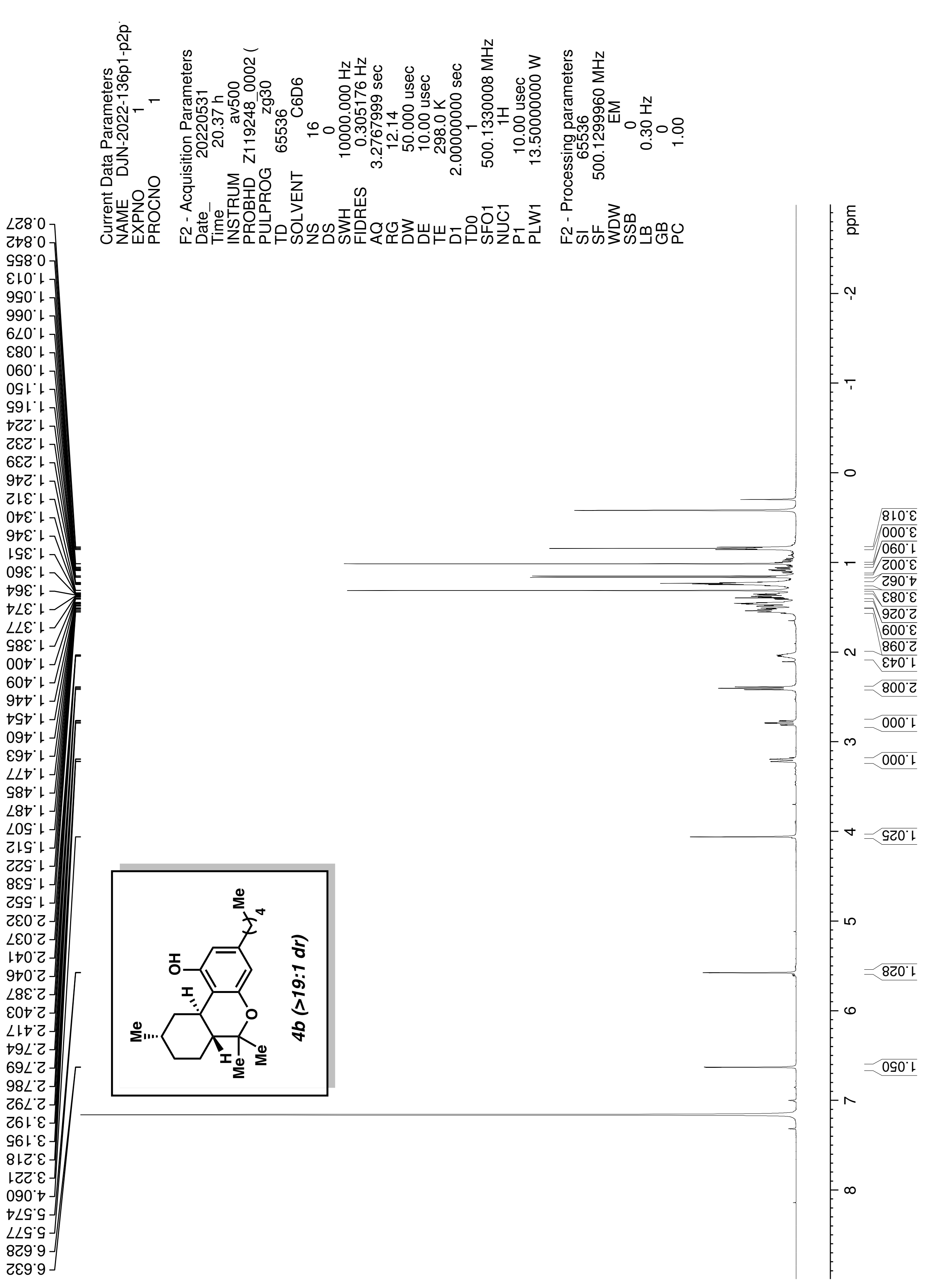
Current Data Parameters
NAME DJN-2022-136p1-p2p
EXPNO 1
PROCNO 1
F2 - Acquisition Parameters
Date_ 20220531
Time 20.37 h
INSTRUM av500
PROBHD Z119248_0002 (
PULPROG zg30
TD 65536
SOLVENT C6D6
NS 16
DS 0
SWH 10000.000 Hz
FIDRES 0.305176 Hz
AQ 3.2767999 sec
RG 12.14
DW 50.000 usec
DE 10.00 usec
TE 298.0 K
D1 2.00000000 sec
TD0 1
SFO1 500.1330008 MHz
NUC1 1H
P1 10.00 usec
PLW1 13.50000000 W
F2 - Processing parameters
SI 65536
SF 500.1299960 MHz
WDW EM
SSB 0
LB 0.30 Hz
GB 0
PC 1.00
0.827 0.842 0.855 1.013 1.056 1.066 1.079 1.083 1.090 1.150 1.165 1.224 1.232 1.239 1.246 1.312 1.340 1.346 1.351 1.360 1.364 1.374 1.377 1.385 1.400 1.409 1.446 1.454 1.460 1.463 1.477 1.485 1.487 1.507 1.512 1.522 1.538 1.552 2.032 2.037 2.041 2.046 2.387 2.403 2.417 2.764 2.769 2.786 2.792 3.192 3.195 3.218 3.221 4.060 5.574 5.577 6.628 6.632
3.018 3.000 1.090 3.002 4.062 3.083 2.026 3.009 2.098 1.043 2.008 1.000 1.000 1.025 1.028 1.050
ppm
OH
H
Me
H
Me
Me
O
Me
4
4b (>19:1 dr)

References:

[1] This research was performed with a Schedule 1 DEA license # RG0538338.
[2] Gaoni, Y.; Mechoulam, R. Hashish – VII The isomerization of cannabidiol to tetrahydrocannabinols. *Tetrahedron* **1946**, *22*, 1481–1488.
[3] Hoffmann, G.; Daniliuc, C. G.; Studer, A. Synthesis of *para* (−)-Δ^8-THC triflate as a building block for the preparation of THC derivatives bearing different side chains. *Org. Lett.* **2019**, *21*, 563–566.
[4] Groves, J. T.; Ma, K. W. Carbon cluster compounds. Generation and reorganization of the homobullvalenyl cation, an 11-fold degenerate species. *J. Am. Chem. Soc.* **1977**, *99*, 4076–4082.
[5] For spectral data of (*9R*)-HHC (**4a**) see: Tietze, L.-F.; von Kiedrowski, G.; Berger, B. Stereo- and regioselective synthesis of enantiomerically pure (+)- and (−)-hexahydrocannabinol by intramolecular cycloaddition. *Angew. Chem. Int. Ed. Engl.*, **1982**, *21*, 221–222.
[6] For spectral data of (*9S*)-HHC (**4b**) see: Archer, R. A.; Boyd, D. B.; Demarco, P. V.; Tyminski, I. J.; Allinger, N. L. Structural studies of cannabinoids. A theoretical and proton magnetic resonance analysis. *J. Am. Chem. Soc.* **1970**, *92*, 5200–5206.
[7] Adams, R. Marihuana active compounds. US Patent No. US2419937A.
[8] Scialdone, M. A. Hydrogenation of cannabis oil. US Patent No. US9694040B2.
[9] Crabtree, R. Iridium compounds in catalysis. *Acc. Chem. Res.* **1979**, *12*, 331–337.
[10] Onyango, E. O.; Kelley, A. R.; Qian, D. C.; Gribble, G. W. Syntheses of 1-Bromo-8-methylnaphthalene and 1-Bromo-5-methylnaphthalene. *J. Org. Chem.* **2015**, *80*, 5970–5972.
[11] Ashby, E. C.; Lin, J. J. Selective reduction of alkenes and alkynes by the reagent lithium aluminum hydride-transition-metal halide. *J. Org. Chem.* **1978**, *43*, 2567–2572.
[12] Kattamuri, P. V.; West, J. G. Hydrogenation of alkenes via cooperative hydrogen atom transfer. *J. Am. Chem. Soc.* **2020**, *142*, 19316−19326.
[13] Spartan 20 version 1.1.3 for Mac, Wavefunction, Inc., Irvine, California, USA, www.wavefun.com.
[14] Chai, J.-D. Head-Gordon, M. Long-range corrected hybrid density functionals with damped atom–atom dispersion corrections. *Phys. Chem. Chem. Phys.* **2008**, *10*, 6615–6620.
[15] C. Y. Legault, CYLview20; Université de Sherbrooke: Quebec, Montreal, Canada, www.cylview.org.
[16] Rinaldi-Carmona, M.; Calandra, B.; Shire, D.; Bouaboula, M.; Oustric, D.; Barth, F.; Casellas, P.; Ferrara, P.; Le Fur, G. Characterization of two cloned human CB_1 cannabinoid receptor isoforms. *J. Pharmacol. Exp. Ther.* **1996**, *278*, 871–878.
[17] GraphPad Prism version 9.5.0 for Mac, GraphPad Software, San Diego, California, USA, www.graphpad.com.
[18] Munro, S.; Thomas, K. L.; Abu-Shaar, M. Molecular characterization of a peripheral receptor for cannabinoids. *Nature* **1993**, *365*, 61–65.
[19] Felder, C. C.; Joyce, K. E.; Briley, E. M.; Mansouri, J.; Mackie, K.; Blond, O.; Lai, Y.; Ma, A. L.; Mitchell, R. L. Comparison of the pharmacology and signal transduction of the human cannabinoid CB_1 and CB_2 receptors. *Mol. Pharmacol.* **1995**, *48*, 443–450.
